# Identification of Novel Physiological Substrates of *Mycobacterium bovis* BCG Protein Kinase G (PknG) by Label-free Quantitative Phosphoproteomics * [Fn FN2]

**DOI:** 10.1074/mcp.RA118.000705

**Published:** 2018-03-16

**Authors:** Kehilwe C. Nakedi, Bridget Calder, Mousumi Banerjee, Alexander Giddey, Andrew J. M. Nel, Shaun Garnett, Jonathan M. Blackburn, Nelson C. Soares

**Affiliations:** From the ‡Division of Chemical & Systems Biology, Department of Integrative Biomedical Sciences, Faculty of Health Sciences, University of Cape Town, South Africa;; §Institute of Infectious Disease & Molecular Medicine, Faculty of Health Sciences, University of Cape Town, South Africa

**Keywords:** Parallel reaction monitoring, Phosphorylation, Serine/Threonine Kinases*, Mass Spectrometry, Post-translational modifications*, Mycobacterium Tuberculosis

## Abstract

Mycobacterial Ser/Thr kinases play a critical role in bacterial physiology and pathogenesis. Linking kinases to the substrates they phosphorylate *in vivo*, thereby elucidating their exact functions, is still a challenge. The aim of this work was to associate protein phosphorylation in mycobacteria with important subsequent macro cellular events by identifying the physiological substrates of PknG in *Mycobacterium bovis* BCG. The study compared the phosphoproteome dynamics during the batch growth of *M. bovis* BCG *versus* the respective PknG knock-out mutant (ΔPknG-BCG) strains. We employed TiO_2_ phosphopeptide enrichment techniques combined with label-free quantitative phosphoproteomics workflow on LC-MS/MS. The comprehensive analysis of label-free data identified 603 phosphopeptides on 307 phosphoproteins with high confidence. Fifty-five phosphopeptides were differentially phosphorylated, of these, 23 phosphopeptides were phosphorylated in *M. bovis* BCG wild-type only and not in the mutant. These were further validated through targeted mass spectrometry assays (PRMs). Kinase-peptide docking studies based on a published crystal structure of PknG in complex with GarA revealed that the majority of identified phosphosites presented docking scores close to that seen in previously described PknG substrates, GarA, and ribosomal protein L13. Six out of the 22 phosphoproteins had higher docking scores than GarA, consistent with the proteins identified here being true PknG substrates. Based on protein functional analysis of the PknG substrates identified, this study confirms that PknG plays an important regulatory role in mycobacterial metabolism, through phosphorylation of ATP binding proteins and enzymes in the TCA cycle. This work also reinforces PknG's regulation of protein translation and folding machinery.

Mycobacterium tuberculosis is the causative agent of tuberculosis (TB), a major health concern worldwide that is responsible for the death of 1.8 million people every year. The World Health Organization (WHO) estimates that one-third of the world′s population is infected with TB, the majority of whom remain latently infected but harbor the risk of developing the active disease later in life (1). It is therefore critical that we generate relevant data aimed at understanding the molecular mechanisms regulating mycobacterial environmental adaptation, and pathogenicity.

In bacteria, protein phosphorylation is a central mechanism for signal transduction of important cellular events, as reviewed previously (2–5). There is increasing supportive evidence indicating that in mycobacteria, Ser/Thr phosphorylation plays a critical role both in the physiology as well as the virulence of this intracellular pathogen (6–13). Interestingly, mycobacteria have an unusually large repertoire of kinases for a bacterium, including 11 two-component system and 11 Ser/Thr protein kinases (STPKs) (PknA-PknL) (14–17). Inference of the possible biological role of these STPKs is critically dependent on knowledge of the substrates they phosphorylate *in vivo*. Therefore, a detailed characterization of physiological STPK substrates is essential to gain a better understanding of the mechanisms by which protein phosphorylation regulates important biological responses in mycobacteria (18).

Of all mycobacterial kinases, Protein Kinase G (PknG) ^1^ is perhaps the best studied yet remains the most intriguing. Unlike the majority of other STPKs, PknG exists as a dimer and because it has no membrane-bound domain, is found in the cytosol of mycobacteria (12, 19, 20). This protein kinase is ubiquitously present in the Mycobacterium genus and it plays different roles in mycobacterial physiology as well as in pathogenicity. For instance, PknG is known to phosphorylate *in vivo* the mycobacterium fork-head associated protein (FHA) GarA (21), which is important in glutamate metabolism (7, 22, 23). Additionally, PknG has been also implicated in the regulation of other important physiological roles such as biofilm formation and redox homeostasis (24) and intrinsic resistance to antibiotics (25). Importantly, experimental evidence shows that, on macrophage infection, inactivation of PknG, either by gene disruption or chemical inhibition, results in the intracellular trafficking and localization of PknG-deficient *Mycobacterium bovis* Bacille Calmette-Guérin (*M. bovis* BCG) into the phagolysosome and ultimately to mycobacterial cell death (12, 13). A recent study also demonstrated its role in the survival of mycobacteria following *in vitro* induction of a nonreplicating state (26). Despite this suggested essential role of PknG in ensuring bacterial environmental adaptation and survival, GarA has until recently been the only known and validated substrate for PknG (7, 23, 27) although a recent study reported that PknG also phosphorylates the 50S ribosomal protein L13 *in vivo* which is important in biofilm formation and maintaining redox homeostasis (24). However, these two substrates alone seem insufficient to account for the phenotype of PknG knock-out strain of *M. tuberculosis*. In addition, our previous work in this area strongly suggests that each Ser/Thr kinases in *M. bovis BCG* phosphorylates multiple substrates (28). Thus, to gain new insights into the protein phosphorylation networks by which PknG mediates important physiological functions, it is crucial that we identify a more comprehensive list of its verified substrates.

Recent advances in mass spectrometry-based methods have revolutionized the research field of phosphoproteomics by allowing global and accurate quantitative analysis of protein events at the phosphopeptide level (29–39). In addition, several studies using quantitative phosphoproteomics have identified hundreds of kinase substrates in varying eukaryotic systems (40–44). More recently, similar quantitative phosphoproteomic analyses have been successfully applied to identify novel substrates of *Bacillus subtilis* Ser/Thr kinase PrkC and phosphatase PrpC (34) as well as of the MAPK Hog1 in yeast (45) and in human cells (40).

Despite the increasing utility of large-scale proteomics for the *in vivo* identification of novel kinase substrates, to our knowledge, such an approach has yet to be employed in the search for mycobacterial STKPs substrates. As a proof of principle, the current study thus presents the first systematic application of large-scale phosphoproteomic analysis to screen for and verify *in vivo* novel physiological substrates of mycobacterial STPKs. This study reveals a new set of protein targets that may help to elucidate the exact physiological role of PknG and its associated phosphorylation networks during adaptive strategies in mycobacteria to changing environments.

## MATERIALS AND METHODS

### 

#### 

##### Bacterial Culture

*M. bovis* BCG reference strain (Pasteur 1172) and *M.bovis* BCG PknG knock-out mutant strain generously donated by Prof Jean Pieters (13) were grown in 7H9 Difco^TM^ Middlebrook liquid media (Becton Dickinson; BD, Franklin Lakes, NJ), supplemented with OADC and Tween 80, to prevent clumping, at 37 °C while shaking. Growth was monitored daily by measuring Optical Density (OD_600_) and the growth curve plotted. Cells were harvested at mid-log (OD ∼ 0.6) by centrifugation for 10 min (4000 × *g*) and washed twice in phosphate buffered saline (PBS) pH 7.4 (Sigma, St Louis, MI). Cell pellets were then snap frozen in liquid nitrogen and stored at −80 °C until needed.

##### Cell Lysates

Lysis buffer (500 mm Tris pH 8, Protease and phosphatase inhibitor mixture tablets (Roche, Mannheim, Germany), lysozyme 5 μg/ml) was added to the frozen pellet and defrosted on ice. Samples were sonicated at 60%, for six times at 30 s intervals, with cooling on ice and centrifuged at 4000 × *g* for 10 min. The supernatant was filter sterilized (0.2 microns) and transferred into a fresh tube. Proteins were precipitated with chloroform/methanol and resuspended in Urea buffer (6 m urea, 2 m thiourea and 10 mm Tris-HCl (pH 8)). Precipitated proteins were quantified with the modified Bradford method (46).

##### Tryptic Digestion

In-solution digestion was carried out on 2.5 mg total protein. Briefly, proteins were denatured with 1 mm dithiothreitol (DTT) for 1 h at room temperature with gentle agitation and alkylated for 1 h in the dark with 5.5 mm iodoacetamide (IAA). Proteins were pre-digested with Lys-C endopeptidase (Wako, Neuss, Germany) for 3 h before being diluted four times with HPLC grade water to a final concentration of 2 m Urea. The diluted sample was then digested overnight with trypsin (1:100 ratio) and digestion quenched with Trifluoroacetic acid (TFA) (Sigma Aldrich).

##### Phosphopeptide Enrichment

A total of 2.5 mg digested peptides were subjected to three rounds of phosphopeptide enrichment by titanium dioxide (TiO_2_) chromatography as previously described (38). Briefly, Acetonitrile (ACN) (Sigma Aldrich) was added to the peptide mixture at a final concentration of 30%. Titasphere TiO_2_ beads (MZ Analysentechnik, Mainz, Germany) in loading buffer (30 mg/ml 2,5-dihydroxybenzoic acid (DHB) (Sigma Aldrich) in 80% ACN) was added to the sample and incubated at room temperature with rotation for one hour. The beads were pelleted, and the decanted supernatant was further incubated with a fresh batch of 5 mg of beads for 30 min and repeated once more. TiO_2_ beads were washed sequentially with 1 ml of wash buffer 1 (30% acetonitrile, 3% trifluoroacetic acid) followed by buffer 2 (80% acetonitrile, 0.1% trifluoroacetic acid). Phosphopeptides were then eluted three times with 50 μl Elution buffer (40% Mass-spec grade NH_4_OH (aq, 25% NH_3_; Sigma Aldrich), 60% acetonitrile) and dried in a speed drying vacuum at room temperature. Phosphopeptides were resuspended in 20 μl Solvent A (2% ACN, 0.1% formic acid) before desalting with in-house Stage-tips.

##### LC-MS/MS Analysis

Cleaned-up phosphopeptides were resuspended in 15 μl of solvent A, of which 5 μl were loaded on to the LC-MS/MS system, *i.e.* HPLC chromatograpy (Dionex Ultimate 3500 RSLC Nano System (Thermo Fisher Scientific, Waltham, MA)) coupled to a Q Exactive mass spectrometer (Thermo Fisher Scientific). The LC delivered a flow rate of 0.4 μl/min to an in-house built 40 cm column (75 μm internal diameter, packed with 3.6 μm particle Aeris C18 beads;Phenomenex) and maintained at 40 °C. Solvent A was 0.1% Formic Acid in HPLC grade water and solvent B was 0.1% Formic Acid in Acetonitrile. Gradient consisted of 1% solvent B for 10 min, then increasing to 6% B over 2 min, and followed by increasing to 35% B over 118 min; washing with 80% B followed. For phosphopeptide quantification, the mass spectrometer acquired spectra in top 10 data-dependent acquisition mode, with precursor ions fragmented by higher-energy collision-induced dissociation (HCD) with a normalized collision energy of 27 and at a resolution of 75,000.

##### Phosphopeptide and Protein Identification

Raw files from the Q Exactive were processed in MaxQuant version 1.5.0.3. MS/MS spectra were searched against the *M.bovis* BCG Pasteur 1172 reference proteome downloaded from UniProt (www.uniprot.com) and containing 3 891 entries (March 2016). Maxquant's built-in Andromeda search algorithm (47) was used to map spectra to the reference proteome with mass tolerance for precursor and fragment ions set at 45 and 20ppm respectively. Estimation of False Discovery Rate was through use of a Target decoy database as per Maxquant settings. Phospho (STY), oxidation of Methionine and N-terminal acetylation were set as variable modifications and carbamidomethylation of cysteine a constant modification. Trypsin and LysC/P were selected as digestion enzymes and two missed cleavages allowed.

##### Experimental Design and Statistical Rationale

Three biological replicates per experiment were processed to obtain statistically significant results. *M. bovis* BCG reference strain and *M.bovis* BCG PknG knock-out mutant strains were used as experiment and control, the logic being that peptides phosphorylated in the presence of PknG and not in the mutant strain would represent possible substrates of PknG. Peak height intensities for each phosphopeptide were used to assess phosphorylation. Phosphopeptides with intensity values in all three replicates in the wild type *M. bovis* BCG and not in the mutant lacking PknG were considered as candidate physiological substrates of mycobacterial PknG. To do a quantitative phosphoproteomic analysis, data were filtered as described by Sharma *et al.*, 2014 (37) and phosphopeptides were normalized by employing the Pair-wise normalization workflow as described by Kauko and colleagues (41), which accounts for the high variance introduced by the phosphopeptide enrichment by adjusting phosphopeptide measurements before and after enrichment. Normalized data were uploaded into Perseus (version 2.3) and a student *t* test with a *p* value cut-off of 0.05 was used to compare the means of the log-transformed (base 2) phosphopeptides between the wild type and PknG knock-out mutant.

##### Validation of Candidate Substrates of PknG

Identified PknG substrates were validated by tier 3 targeted mass spectrometry workflow in accordance with MCP guidelines. Briefly, the discovery phosphoproteomic data were used to identify peptides with confidently localized phosphosites (localization probability of ≥0.75) that were present in the *M.bovis* BCG wild type and absent in all three replicates of the PknG knock-out mutant. Fourteen of these peptides, derived from 7 proteins, were selected to validate the phosphoproteomic data. A spectral library was generated using the discovery phosphoproteomic data in Skyline (version 3.6.0.10162), with the best representative spectrum for each identified peptide and phosphopeptide. Retention times were calculated based on the average retention time observed in the discovery phosphoproteomic analysis. An isolation list was generated with a 10-min retention time window around each peptide's calculated retention time. This isolation list was used to carry out a 2-plex scheduled Parallel Reaction Monitoring (PRM) analysis with 100 ms injection time and a total cycle time of 2 s on a Q Exactive hybrid Orbitrap mass spectrometer. The AGC target was set to 5e^6^ maximum, and a 2 *m*/*z* mass error window was allowed. Targeted MS2 data were acquired at a resolution of 35000. The chromatography setup was identical to that of the discovery phosphoproteomic analysis. The resulting PRM data were analyzed in Skyline with the background *M. bovis* BCG database obtained from UniProt (www.uniprot.com). The spectral library was used to confirm the identity of the targeted peptides. Raw PRM data were uploaded to panorama online database available at https://panoramaweb.org/labkey/D3nPgL.url.

##### Kinase-substrate Interactions

To understand the kinase-substrate interaction of the target peptides, a structure-based modeling study was undertaken. The target peptides were modeled and docked into an existing ADP-bound PknG structure (PDB id 4Y0X) using CABS-dock web server (http://biocomp.chem.uw.edu.pl/CABSdock) to identify each peptide's binding site. The server performed a simulation search (using a set of 10,000 models) for the binding site, allowing for full flexibility of the peptide. A small fluctuation of the receptor backbone during binding was allowed. A final model was selected using a two-step procedure of (1) initial filtering of lowest binding energy model and (2) k-medoids clustering. The best peptide binding was chosen from the lowest binding energy which is the sum of Lenard-Jones and Coulombic potential functions from popularly used force fields (48, 49). Figures were generated using PyMol (www.pymol.org).

## RESULTS

### 

#### 

##### Growth Monitoring of Bacterial Strains

**Fig. 1.**
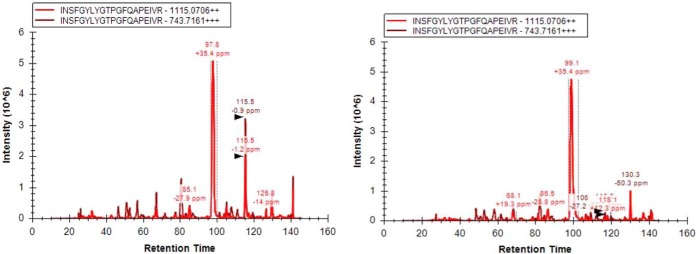
**Targeted PknG peptide (INSFGYLYG) identified exclusively in the Wt *M. bovis* BCG and not in the knock-out mutant.**

*M. bovis* BCG Pasteur 1172 reference strain and *M. bovis* BCG ΔPknG knock-out mutant (13) were cultured in nutrient-rich 7H9 broth media and growth rates were compared (supplemental Fig. S1*A*) shows growth curves monitored by measuring optical density (OD_600_) of the culture over time. The PknG knock-out mutant grew at the same rate as the wild-type strain, in line with previous studies (10, 12). We then validated that the knock-out mutant was indeed lacking PknG by targeted mass spectrometry (MS). PknG peptides identified by discovery mass spectrometry were quantified by PRM in both strains as illustrated in Fig. 1. PknG peptides were unambiguously identified in the wild-type reference strain but not in the ΔPknG knock-out mutant.

##### Identification of Physiological Substrates for PknG

We employed large-scale label-free quantitative phosphoproteomics to study global phosphorylation dynamics associated with *M. bovis* BCG PknG and to identify *in vivo* physiological substrates for this kinase in actively growing bacterial cells in rich media, to try to understand the regulatory role it plays while in the cytosol. We compared the phosphoproteome of wild-type and PknG knock-out strains of *M.bovis* BCG, in biological triplicate. The experimental workflow is shown in supplemental Fig. S1*B*. Exponentially growing cells of *M. bovis* BCG and PknG knock-out mutants were lysed and proteins precipitated and digested with trypsin. Phosphopeptides were enriched with TiO_2_ after proteolysis, followed by liquid chromatography-tandem mass spectrometry (LC-MS/MS). The mass spectrometry proteomics data have been deposited to the ProteomeXchange Consortium (http://proteomecentral.proteomexchange.org) via the PRIDE partner repository with the dataset identifier PXD006473 (50).

**Fig. 2.**
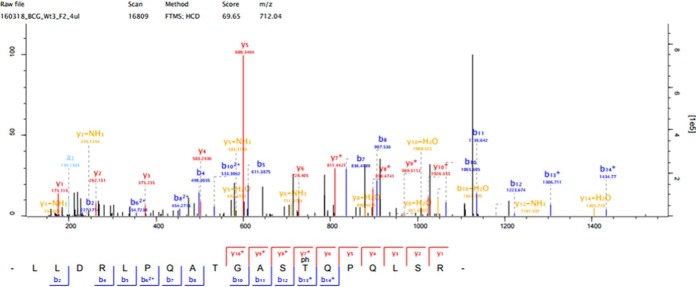
**Fragmentation spectra of the phosphopeptide showing both b- and y-ions of the phosphorylated peptide from one of the candidate substrate Chaperone protein ClpB**.

A total of 829 phosphosites (p-sites) were identified. After removing known contaminants and applying filters (37) (localization probability ≥0.75, PEP <0.01, FDR <0.05, Score >40, Delta Score >8), 603 highly confident phosphopeptides were identified on 307 phosphoproteins and were further analyzed (supplemental Table S1). Phosphorylated residues were also manually validated by looking at the fragmentation spectra using the Maxquant feature “Viewer” as shown in Fig. 2 (See also supplemental Fig. S2). Approximately 93% of phosphopeptides identified were singly phosphorylated, whereas 7% were multiply phosphorylated. The majority of these phosphopeptides were phosphorylated on Thr (64.5%) and Ser (32.84%) and only a small percentage on Tyr (2.65%). These distributions of p-sites are consistent with our data on the phosphoproteome of *M. bovis* BCG (38), but notably, we have increased the phosphoproteome coverage by 100% from 442 to 829 p-sites.

##### Global Phosphorylation Changes Resulting from PknG Gene Knock-out

To correlate the global changes at the phosphorylation level because of PknG gene knock-out, we performed label-free quantitative phosphoproteomic analysis, where we only considered phosphopeptides that were identified in at least two replicates per experiment. Because of the variability introduced by the TiO_2_ enrichment step, we normalized the phosphopeptides from the enriched samples by those that could be detected in the unenriched samples according to a previously published workflow (41). After normalization and filtering, the differential phosphorylation was determined by a two-sample student's *t* test with a significance cut-off *p* value of 0.05. A total of 31 phosphopeptides were significantly differentially regulated between the wild-type and ΔPknG *M. bovis* BCG strains (Table I). Functional analyses of these differentially phosphorylated proteins showed that they were associated with the biosynthesis of macromolecules, two-component system proteins and protein kinase A. For candidate substrates of PknG, we considered those phosphopeptides that were detected in all replicates of the wild-type *M. bovis* BCG but that were absent in all the replicates of *M. bovis* BCG PknG knock-out mutant. The 23 p-sites on 22 proteins identified as *bona fide* candidate substrates of *M. bovis* BCG PknG are summarized in Table II.

**Table I:** Differentially phosphorylated proteins between wild type M. bovis BCC and PknG knock-out mutant

Gene names	Protein names	P-sites	Localization probabilities	-Log Student's T-test *p* value
mtrA	Two-component sensory transduction transcriptional regulatory protein	T213	1	1.97384
cfp29	29 kDa antigen	T47	0.991988	2.08032
psk13	Polyketide synthase pks13	T569	1	1.85385
ctaE	Probable cytochrome C oxidase (Subunit III) ctaE	T7	0.993445	3.94207
PknA	Transmembrane serine/threonine-protein kinase A	T224	1	2.04384
BCG_1592	Pseudouridine synthase	S56	1	1.41308
35kd_ag	Conserved 35 kDa alanine-rich protein	S168	0.971359	1.92842
ansP1	Probable l-asparagine permease ansP1	T474	1	2.59646
rpsQ	30S ribosomal protein S17	T123	0.998357	1.60323
atpFH	ATP synthase subunit b-delta	T78	1	1.59266
BCG_0735	Probable membrane protein	S74	1	1.99181
groL1	60 kDa chaperonin 1	T435	1	1.52333
BCG_134	Uncharacterized protein	S107	0.999885	1.30694
moeW	Possible molybdopterin biosynthesis protein moeW	S107	1	1.48633
infC	Translation initiation factor IF-3	T5	0.932525	1.69633
rpsD	30S ribosomal protein S4	T147	0.999988	1.49155
BCG_0330	Probable conserved transmembrane protein	T4	0.887233	1.64658
BCG_1746	Probable conserved transmembrane protein	T394	0.967662	1.67599
BCG_1664	Probable two-component system transcriptional regulator	S146	0.983817	1.92198
tatA	Sec-independent protein translocase protein TatA	T60	0.999904	1.69263
BCG_1812C	Hypothetical integral membrane protein	T481	0.854165	1.68682
tatA	Sec-independent protein translocase protein TatA	T58	0.999999	1.70645
BCG_2246	Uncharacterized protein	T153	0.984392	1.72215
BCG_0215	Probable conserved MCE associated membrane protein	T62	0.997238	1.4332
BCG_0421c	Possible conserved secreted protein	T210	1	1.31913
PknA	Transmembrane serine/threonine-protein kinase A pknA	S299	1	1.59262
BCG_0194	Probable transcriptional regulatory protein (Possibly tetR-family)	T5	0.983476	1.55204
leuA	2-isopropylmalate synthase	T595	0.999649	1.37648
BCG_0875c	Uncharacterized protein	S82	1	1.94697
metE	5-methyltetrahydropteroyltriglutamate–homocysteine methyltransferase	S95	0.999759	1.3025
mas	Probable multifunctional mycocerosic acid synthase membrane-associated mas	S2111	1	2.70602

**Table II:** Candidate substrates of PknG only phosphorylated in wild type M. bovis BCG. Known PknG substrates GarA and L13 were not identified in this study, however, were included in the analysis for comparison purposes

Protein Names	UniProt IDs	P-sites	Localization Probability	Subcellular Localization	Free Binding Energy
50S ribosomal protein L2	A1KGI5	S32	1	Cytoplasmic	−2915.29
Chaperone protein ClpB	A0A0H3M7W9	T79	0.89	Cytoplasmic	−2708.49
Probable conserved membrane protein	A0A0H3M0X2	S19	0.99	Cytoplasmic	−2704.67
Uncharacterized protein	A0A0H3M8P9	T12	0.93	Cytoplasmic	−2448.17
Antitoxin	A0A0H3M1S6	T152	1	Cytoplasmic	−2401.27
GarA					−2321.83
Uncharacterized protein	A0A0H3M6A1	S2	0.99	Cytoplasmic	−2250.28
L13					−2226.24
Uncharacterized protein	A0A0H3M4P0	S11	0.99	Integral membrane	−2209.59
metE	A1KHS4	S713	0.99	Cytoplasmic	−2022.95
ATP synthase subunit beta	A1KI98	S16	0.79	Cytoplasmic	−1856.33
Uncharacterized protein	A0A0H3M751	Y382	0.99	Cytoplasmic	−1829.71
ispG	A1KML3	S387	0.97	Cytoplasmic	−1795.16
Uncharacterized protein	A0A0H3MC79	S277	0.78	Cytoplasmic	−1767.57
30S ribosomal protein S16	A1KMQ3	S162	0.99	Cytoplasmic	−1735.73
proline and threonine-rich protein	A0A0H3MAA7	S403	0.98	Integral membrane	−1674.22
Uncharacterized protein	A0A0H3M751	T371	0.99	Cytoplasmic	−1577.51
DNA gyrase subunit A	A0A0G2Q9F8	S263	1	Cytoplasmic	−1425.1
Uncharacterized protein	A0A0H3M7J9	T7	0.99	Cytoplasmic	−1359.11
Antitoxin	A0A0H3MAL0	T109	0.95	Attached to membrane by lipid anchor	−1350.54
Uncharacterized protein	A1KI28	S117	0.83	Integral membrane protein	−1301.16
Uncharacterized protein	A0A0H3M2H1	T25	0.77	Cytoplasmic	−1247.69
RNA polymerase-binding protein RbpA	A0A0H3M6B6	T18	0.95	Cytoplasmic	−1170.72
Malate dehydrogenase	A1KI28	S238/117	0.92	Integral Membrane Protein	−1064.63
Chaperone protein DnaK	A1KFH2	T391	0.99	Integral Membrane Protein	−725.77

The quantification of phosphorylated proteins is important to our understanding of the resultant biological effects. To ensure that these significantly dysregulated phosphopeptides were not because of changes at the proteome level, we performed a quantitative proteome analysis parallel to the phosphoproteomic workflow. All proteomic analyses were carried out using Perseus version 2.3. We considered all proteins with intensity values reported in all three replicates in each experimental group. Logarithmic means (base 2) of the wild type and knock-out mutants were compared using a student's *t* test with a cut-off value of *p* = 0.05 and with Benjamini-Hochberg multiple testing correction. Of all the differentially regulated proteins (supplemental Table S1), candidate substrates did not change at the proteomic level, except two (MetE and ClpB, both more abundant in the wild type strain). The lack of change at the protein level is a strong indicator that regulation is at the phosphorylation level and not because of expression levels of the phosphorylated proteins.

##### Validation of Candidate Substrates by Targeted Mass Spectrometry

**Fig. 3.**
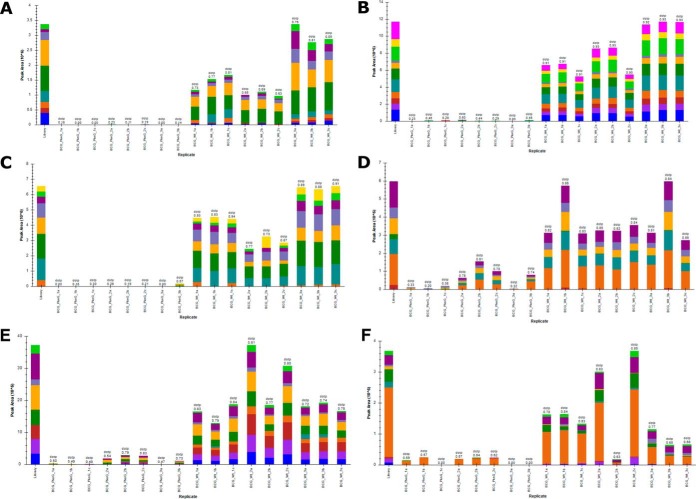
**Validation of identified phosphopeptides by targeted PRM's.** *A–C*, shows phosphopeptides that were exclusively identified in the Wild-type *M.bovis* BCG and not in the PknG knock-out mutant, whereas *D–F*, shows differential phosphorylation of the substrates of PknG.

To further validate the candidate substrates identified by this phosphoproteomic workflow, we employed targeted mass spectrometry (PRMs). An isolation list for all the peptides that were detected in the wild-type but not in the mutant was created. We selected six of the candidate substrates to be validated by PRMs and Fig. 3 shows that these phosphopeptides were unambiguously verified to be absent in the PknG mutant but present in the wild-type (Fig. 3). In the remaining cases, we observed a decrease in the amount of the phosphorylated peptide in the PknG knock-out mutant compared with the wild-type (Fig. 3*D*–3*F*). We designed PRM assays to detect both modified (phospho Ser/Thr/Tyr) and unmodified versions of each peptide in the isolation list. We observed a substantial shift in retention times during these experiments, which is a technical issue and resulted in the truncation of some extracted PRM chromatograms, so some candidate substrates could not be confidently and unambiguously validated by this targeted approach.

##### Kinase-substrate Interactions

**Fig. 4.**
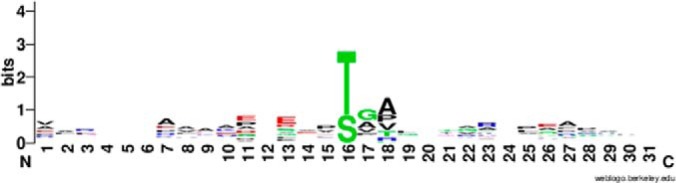
**Phosphorylation site motif analysis generated using IceLogo.** Showing overrepresented amino acids around the phosphorylation site.

To determine if there are any preferential motifs for PknG substrate specificity, we used iceLogo (www.weblogo.berkely.edu) to visualize overrepresented amino acids flanking the phosphorylation sites of candidate substrates that indicate interaction with this kinase (Fig. 4). This sequence alignment shows a major feature around the (Ser/Thr) phosphorylation site, specifically an over-representation of glycine (G) at position +1 and alanine (A) or proline (P) at position +2. There is also a strong selection of hydrophobic amino acids at these positions adjacent the phosphorylation site. Enrichment for glutamic acid (E), occurred at positions −3 and −5.

**Fig. 5.**
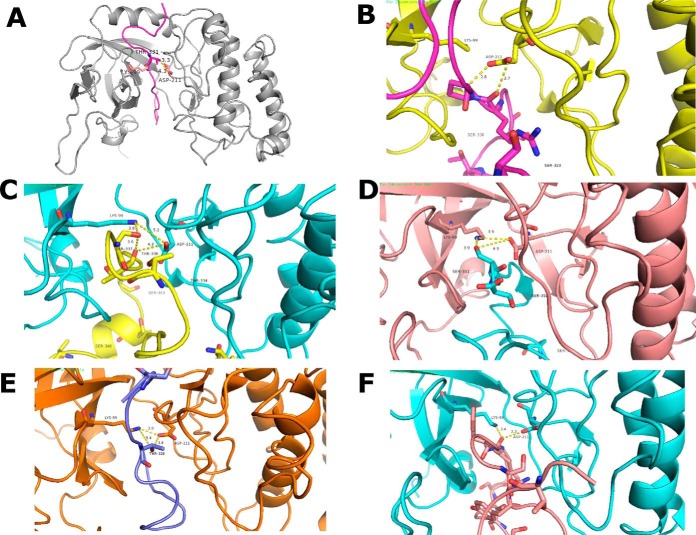
***A*, PknG binding with (PDB ID: 4Y0X) with GarA.** PknG chain in gray color and GarA peptide in pink. The threonine residue near to the catalytic residues shown as ball and stick model. The γ- hydroxyl group is within hydrogen bonding distance of carboxyl group of Asp211. *B–E*, shows the interaction of the high confidence substrates with the catalytic core of PknG. *B*, 50S ribosomal protein L2; *C*, A0A0H3M0X2_MYCBP probable conserved membrane protein; *D*, chaperone protein ClpB; *E*, A0A0H3M6A1_MYCBP uncharacterized protein; *F*, A0A0H3MAA7_MYCBP conserved hypothetical proline and threonine rich protein.

The lack of specific motif characteristic of eukaryotic kinases prompted us to investigate the structural basis for the kinase-substrate interaction, using the CABS dock software to simulate interactions of candidate peptides into the catalytic groove of the protein kinase, and with an energy-based optimization that allows for flexibility. Visual analysis was carried out using PyMol version 1.3 software. The crystal structure of PknG in complex with ADP (51) was used as a reference to model the kinase-substrate interactions (PDB ID: 4Y0X). Fig. 5*A* shows the modeling simulation of the validated substrate of PknG GarA kinase-substrates complexes; the highly confident candidate substrates identified in this study interacting with PknG are shown in Fig. 5*B*–5*F*. Visualization of the interactions shows a unique hydrogen bond between the carboxyl group of Asp211 of the catalytic side of PknG and the γ- hydroxyl group of phosphorylated residues in the candidate substrates. The distance between the hydrogen bonds was within the acceptable distance for protein-peptide interactions, ranging from 2.9 Å for 50S Ribosomal Protein L2, 4.3 Å for Chaperon Protein ClpB, and 3.9 Å for a probable conserved membrane protein (A0A0H3M0X2). These results suggest a similar substrate/kinase interaction to that observed for the GarA interaction with PknG, as well as for the recently proposed substrate of PknG (24), ribosomal L13. The Gibbs free binding energies were predicted and compared with GarA, a previously identified and validated substrate of PknG, as a measure of confidence for binding specificity (Table II). From the 23 candidate substrates, five had lower (better) free binding energy than GarA (-2.3Kcal.mol^−1^), ranging between −2.4Kcal.mol^−1^ for Antitoxin protein and −2.9Kcal.mol^−1^ for 50S Ribosomal Protein L2, suggesting that these peptides are true interactors of PknG.

##### Functional and Spatial Relationships of Candidate Substrates

**Fig. 6.**
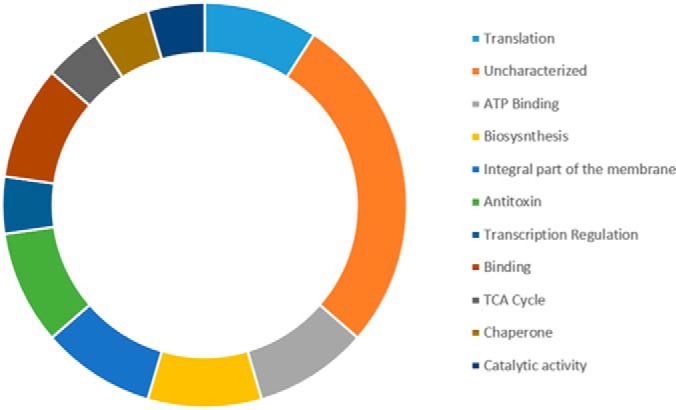
**Functional categories of all identified candidate substrates of *M. bovis* BCG PknG**. The most represented functional categories are Translation, ATP Binding, Biosynthesis, and Antitoxin.

Identified candidate substrates of PknG were involved in diverse cellular functions. We used the Uniprot online database to classify the functional categories for each substrate. Fig. 6 shows a plot of all the major functional groups represented in the discovery MS analysis. PknG thus appear to regulate a wide variety of biological processes including transcription regulation, metal binding and DNA binding. The most overrepresented categories were ATP binding and ATP synthesis, strongly suggesting that PknG plays an important role in ATP regulation. *M. bovis* BCG has a significant number of proteins that are uncharacterized, and in our data, 8 of the PknG substrates were of unknown function, although a sequence alignment showed that one of these proteins (UniProt ID: A0A0H3M751) is an FHA domain containing protein which has been shown to bind phosphopeptides (52). As GarA also contains the FHA domain, PknG, like other kinases, has an affinity for FHA domain containing proteins. Another domain enriched among these uncharacterized proteins was the ATPase domain (UniProt ID: A0A0H3MC79). PknG is a cytosolic kinase and it has been experimentally shown that these kinases phosphorylate substrates in the same subcellular sites they are found *in vivo* (53). We used the TB-pred online tool (www.tbpred.com) to identify the subcellular locations of the candidate substrates, as shown in Table II. These colocalization data show that most of the candidate substrates are cytosolic, which adds another layer of confidence that these are indeed *bona fide* substrates of PknG in actively growing cultures.

## DISCUSSION

### 

#### 

##### Large-scale LC-MS/MS is Applicable to Search for STPK In Vivo Substrates

Several previous studies have examined phosphorylation by mycobacterial STPKs *in vitro* and have identified a considerable number of putative substrates based on these assays (21, 54–60). However, a recognized limitation of *in vitro* assays is the high enzyme: substrate ratios (up to 1:1) required to detect phosphorylation (7, 27). This means that, in many cases, the biological selectivity of the kinase is probably lost in *in vitro* assays. Thus, the principal conundrum lies in reconstructing accurate *in vivo* phosphorylation networks, linking the kinases to their physiological substrates.

There has been some controversy around the role of PknG during growth in liquid culture. The essential role of PknG in culture is evident based on the growth defects of the *M. tuberculosis* ΔPknG knock-out mutant when compared with the wild-type in nutrient-rich media (22, 61). These studies found that the *M. tuberculosis* mutant lacking the PknG gene had significant growth defect when cultured in different growth media. It is still unclear why these growth defects are evident in *M. tuberculosis* but not in *M. bovis* BCG (10, 12, 13), as confirmed in the present work. The lack of obvious growth phenotypic differences in *M. bovis* BCG encouraged us to investigate the underlying phosphoproteomic changes regulated by PknG to determine if the kinase plays a redundant role or would still target a specific group of proteins. As proof of principle, the current study presents the first systematic application of large-scale phosphoproteomic analysis to screen for *in vivo* novel mycobacterial STPK physiological substrates.

Previous mass spectrometry-based phosphoproteomics studies have identified several hundred phosphopeptides/proteins in different mycobacterial species including *M. bovis* BCG ((30, 38, 39, 62). Nevertheless, the physiological significance of these findings remains unclear, especially given the suspected promiscuity of STPKs toward different substrates (63). In the present analysis, more than 600 phosphorylation events were quantified and of these, <15% showed a differential phosphorylation between wild type *M. bovis* BCG *versus* ΔPknG. This indicates that under the studied conditions there is a reduced subset of proteins that are potentially regulated by PknG, pointing toward kinase/substrate selectivity, rather than kinase promiscuity. Furthermore, our results clearly identified two categories of differentially regulated phosphopeptides: those that appeared with higher phosphorylation levels in wild type *M. bovis* BCG than in ΔPknG strain and those phosphopeptides that were detected exclusively in wild type (both through discovery and PRM analysis) but not in the ΔPknG strain with high reproducibility. In the first category, it could be argued that those proteins may not be directly phosphorylated by PknG, but instead by other protein kinase(s) whose activity was itself affected by the absence of PknG (for example PknA that appeared among those being differentially phosphorylated). However, the second category of differential phosphorylation events seems more likely to depend entirely on the presence of PknG, although even here, the possibility that these proteins are phosphorylated by other STPKs cannot be completely ruled out. This second category of phosphoproteins can thus be initially considered as potential direct PknG substrates for further validation.

The use of targeted MS to follow up phosphorylation events in this study increases the precision and accuracy of the identification of site-specific phosphorylation and proves that targeted mass spectrometry is valuable in phosphoproteomic studies especially where antibodies are not a viable option to validate individual phosphorylation events. We have previously applied PRM to validate phosphopeptides identified by DDA (69) and we used the same methodology in this work to validate the identity and localization of candidate substrates. SRMs and MRMs have been previously used to validate phosphosites in mass-spectrometry-based phosphoproteomic datasets (64–68). By selecting the Ser/Thr/Tyr residue(s) for monitoring it is possible to directly assess the localization of the phosphorylation event. Usefully, PRM differs from SRM in that all generated fragment ions are monitored simultaneously and so a more confident assignment is possible in label-free PRM p-site validation than in SRM.

Importantly, the quantitative mass spectrometry-based phosphoproteomic strategy used here allows for confident identification and localization of the phosphorylated sites (See supplemental Table S1). This information then allows further analysis to predict/assess the probability of the identified phosphoproteins being phosphorylated by protein kinase in question. As discussed below, here employed *in silico* analysis to further narrow down the initial list of phosphoproteins to a confident list of potential PknG substrates.

##### PknG Protein-Peptide Docking Interactions Suggest the Basis of Specificity

Identification of targeted proteins and the precise phosphorylated peptides provides a unique opportunity to extend our understanding of molecular interactions between kinases and their substrates. Both known PknG substrates GarA and ribosomal L13 have a nonpolar amino acid two positions before phosphorylatable Thr (Val19 and Gly10 respectively). Similarly, in this study, PknG *in vitro* phosphorylated sites that have nonpolar amino acids two positions before the phosphorylatable Thr. This common feature at the −2 peptide residue is suggested to be stabilized by van der Waals interactions within a small pocket comprising hydrophobic groups (51). Interestingly, the sequence alignment of the candidate substrates presented a predominant presence of nonpolar amino acids at +1 and +2 residues from the phosphorylation site. This configuration resembles the mode of the binding of substrates to eukaryotic protein kinases, in which the position of phosphorylation is secured by + a 1 loop that accommodates the neighboring (*p* + 1) residue. Lee and colleagues found, through sequence alignment and site-specific mutagenesis, that the tyrosine phosphatase Src kinase (CSK) has a high affinity for binding substrates with nonpolar residues immediately following the binding site (70). Our results are thereby in line with the reported extensive nonpolar and polar interactions between the AX20017 inhibitor and PknG, where the inhibitor is bound deep within a hydrophobic PknG binding pocket (12, 20). Finally, the alignment indicated a selection for glutamic acid (E) at positions −3 and −5. Prisic and coworkers demonstrated that acidic residues from −2 to −5 increased the susceptibility for phosphorylation of proteins by most of the *M. tuberculosis* STPKs and that there is an apparent preference for Glu or Asp at this position among the STPKs (62). In agreement with Prisic's work, our results clearly indicate that PknG shows selectivity for Glu at those positions, however, the molecular role of this residue in phosphorylation by PknG requires further study.

The pattern observed in the peptide sequences, *i.e.* lack of specific sequence motifs, is possibly indicative of the low specificity yet high affinity of PknG to its substrates. This supports earlier suggestions that STPKs substrates could be better identified as interacting partners rather than a consensus sequencing surrounding the phosphorylation site (51). Further, our docking analysis showed that from the 23 candidate substrates, five had lower (better) free binding energy than GarA (−2.3 Kcal.mol^−1^), ranging between −2.4 Kcal.mol^−1^ for Antitoxin protein and −2.9 Kcal.mol^−1^ for 50S Ribosomal Protein L2, supporting the notion that these peptides interact with PknG. Of note the five most stable phosphopeptides contain a Pro in position +2 or +3 from the phosphorylation site, suggesting that these positions could be stabilized by van der Waals interactions within a hydrophobic PknG binding pocket. This would imply that in such cases the substrate interaction with the PknG catalytic site occurs in an opposite orientation from that observed in GarA in which −2 or −3 positions are the suggested stabilizing positions.

This study has demonstrated the utility of mass-spectrometry-based phosphoproteomics in the screening for STPK substrates by comparing the global phosphoproteome of an *in vivo* system with and without PknG. The consistent detection of specific phosphopeptides in all three biological replicates of the wild type and their respective absence from ΔPknG samples argues that at least these peptides are likely to be specifically targeted by PknG with a biological purpose. It can, therefore, be reasoned that the list of potential PknG substrates identified here are preferentially phosphorylated by PknG and the extent of substrate phosphorylation would then drive a resultant cellular response.

Our protein-functional analysis revealed several potential PknG substrates related to protein translation (Fig. 6). Noteworthy among these is the presence of two ribosomal proteins, including 50S ribosomal protein L2, that presented maximum localization probability value (1.0) and which had the lowest free binding energy in docking models. This indicates 50S ribosomal protein L2 as a strong candidate substrate of PknG and it is in line with earlier reports that identified ribosomal proteins as substrates of PknG (24). Interesting proteins that are involved in protein translation included two antitoxin proteins: RelB-like antitoxin protein and VapB. Toxin-antitoxin (TA) modules have been described as potential regulators of growth in *M. tuberculosis* and other bacteria, as reviewed by Page and Peti (71). RelB-like and VapB are part of type II toxin-antitoxin family in which the toxin normally inhibits cell growth, whereas the antitoxin neutralizes the activity of toxin by forming a tight TA complex (72). Toxin-antitoxin systems are often described as stress response mechanisms and during batch cell culture might be expected to act later in the growth curve when cells transition into a stationery phase of growth. However, our observation indicates that antitoxins are present in exponentially growing mycobacteria cells. It is therefore tempting to picture a scenario where phosphorylation of antitoxins by PknG is part of surveillance mechanism that regulates binding of these antitoxins to their cognate toxins. This suggests in turn that PknG kinase activity may play a role in coordinating mycobacterial adaptation to changing environmental conditions, including dormancy (26).

Finally, GarA and ribosomal protein L13 previously identified as substrates of PknG were not detected in our assay. This apparent contradictory result can be easily explained by the fact that here the cells were harvested during exponential phase, whereas previously both GarA and ribosomal proteins were seen to be phosphorylated by PknG in cells under stressed and/or nutrient limiting conditions, as well as high kinase: substrate ratios in *in vitro* assays (23, 24, 27). It would therefore be interesting to extend our search for PknG substrates to include conditions that limit cell division and growth.

## CONCLUSIONS

We have carried out a high throughput mass spectrometry-based phosphoproteomics analysis to characterize substrates of mycobacterial PknG in actively growing *M. bovis* BCG cells. Our results were further validated by in silico docking experiments as well as by targeted mass spectrometry. This described here workflow is robust and should represent a valuable resource to further characterize STPK's substrates in different growth conditions to enhance to our understanding of these important regulatory proteins.

## DATA AVAILABILITY

Mass spectrometry phosphoproteomics data have been deposited to the ProteomeXchange Consortium via the PRIDE partner repository with the dataset identifier: PXD006473. PRM data have been deposited to Panorama, panoramaweb. Link: https://panoramaweb.org/D3nPgL.url. Identifier: Experiment ID:399; Title: PknG physiological substrates.

## Supplementary Material

Supplemental Data

## Footnotes

^1^ The abbreviations used are:

PknG: Protein Kinase G
DHB: 2,5-dihydroxybenzoic acid
FHA: Fork-head association
IAA: Iodoacetamide
*M. bovis* BCG: *Mycobacterium bovis* Bacille Calmette-Guérin
M. smegmatis: Mycobacterium smegmatis
M. tuberculosis: Mycobacterium tuberculosis
OD: Optical Density
PBS: Phosphate Buffered Saline
PRM: Parallel Reaction Monitoring
p-sites: Phosphosites
STPK: Serine/Threonine Protein Kinases
TB: Tuberculosis
TFA: Trifluoroacetic acid
TiO_2_: Titanium dioxide
WHO: World Health Organization
Wt: Wild-type.
