# Supplementary material for: Identification of Novel Physiological Substrates of Mycobacterium bovis BCG Protein Kinase G (PknG) by Label-free Quantitative Phosphoproteomics
Source: Mol Cell Proteomics. 2018 Mar 16;17(7):1365–77. doi: 10.1074/mcp.RA118.000705 (PMC6030727; doi:10.1074/mcp.RA118.000705)
Supplement: Supplemental Data [file supp_17_7_1365__index.html]

Identification of novel physiological substrates of Mycobacterium Bovis BCG Protein Kinase G (PknG) by label-free quantitative phosphoproteomics — Candidate substrates of mycobacterial PknG — Identification of Novel Physiological Substrates of Mycobacterium bovis BCG Protein Kinase G (PknG) by Label-free Quantitative Phosphoproteomics — Supplemental Data 

# Identification of Novel Physiological Substrates of *Mycobacterium bovis* BCG Protein Kinase G (PknG) by Label-free Quantitative Phosphoproteomics

## Supplemental Data

- List of figures and tables - List of figures and tables
- Suppplemetary Figure 1 and 2 - Figure 1 (a): Growth curves Figure 1 (b): Experimental workflow Figure 2: Fragmentation spectra of phosphopeptides
- Supplementary table 1 - List of all identified phosphopeptides, Differentially regulated proteins and phosphopeptides normalization strategy
- Supplementary\_Table\_2 - Evidence file for all identified peptides
- Supplementary\_Table\_3 - PRM\_Peptide\_Transition List
