# Supplementary material for: Identification of Novel Physiological Substrates of Mycobacterium bovis BCG Protein Kinase G (PknG) by Label-free Quantitative Phosphoproteomics
Source: Mol Cell Proteomics. 2018 Mar 16;17(7):1365–77. doi: 10.1074/mcp.RA118.000705 (PMC6030727; doi:10.1074/mcp.RA118.000705)
Supplement: Supplemental Data [file supp_RA118.000705_136253_0_art_file_82107_p4lpdm.pdf]

List of figures:

Figure 1: Targeted PknG peptide (INSFGYLYG) identified exclusively in the wild type *M. bovis* BCG and not in the knock-out mutant.

Figure 2. Fragmentation spectra of the phosphopeptide showing both b- and y-ions of the phosphorylated peptide from one of the candidate substrate Chaperone protein ClpB

Figure 3. Validation of identified phosphopeptides by targeted PRMs. Panels **(A-C)** show phosphopeptides that were exclusively identified in the wild type *M. bovis* BCG and not in the PknG knock-out mutant, whilst panel **(D-F)** show differential phosphorylation of the substrates of PknG.

Figure 4: Phosphorylation site motif analysis generated using IceLogo, showing over-represented amino acids around the phosphorylation site.

Figure 5: (A) PknG binding to (PDB ID: 4Y0X) GarA. PknG chain is shown in gray colour and GarA peptide in pink. The threonine residue near to the catalytic residues is shown as ball-and-stick model. The γ- hydroxyl group is within hydrogen bonding distance of carboxyl group of Asp211. **(B-E)** shows the interaction of the high confidence substrates with the catalytic core of PknG.

Figure 6: Functional categories of all identified candidate substrates of *M. bovis* BCG PknG. The most represented functional categories are Translation, ATP Binding, Biosynthesis, and Antitoxin.

List of Tables:

Table 1: Differentially phosphorylated proteins between wild type *M. bovis* BCC and PknG knock-out mutant

Table 2: Candidate substrates of PknG only phosphorylated in wild type *M. bovis* BCG. Known PknG substrates GarA and L13 were not identified in this study, however, were included in the analysis for comparison purposes.

Supplementary table 1: List of all identified phosphopeptides, Differentially regulated proteins and phosphopeptides normalization strategy

Supplementary Figure 1: (a) Growth curves measured by OD_{600} of the *M. bovis* BCG strains used in this study. (b) Experimental procedures in this study. Briefly, exponentially growing cells of *Mycobacterium bovis* BCG Wt and PknG knock-out mutants were harvested, lysed. Proteins were digested in solution after precipitation with Methanol/chloroform. Three rounds of TiO_2 enrichment of phosphopeptides was carried out and measured on the QE. Data processing and analysis were done on Maxquant and R-studio. Targeted MS on peptides of interest was analysed on skyline

Supplementary Figure 2: Manual validation of phosphosite of all the candidate substrates of PknG through Maxquant "Viewer"
