# Supplementary material for: Identification of Novel Physiological Substrates of Mycobacterium bovis BCG Protein Kinase G (PknG) by Label-free Quantitative Phosphoproteomics
Source: Mol Cell Proteomics. 2018 Mar 16;17(7):1365–77. doi: 10.1074/mcp.RA118.000705 (PMC6030727; doi:10.1074/mcp.RA118.000705)

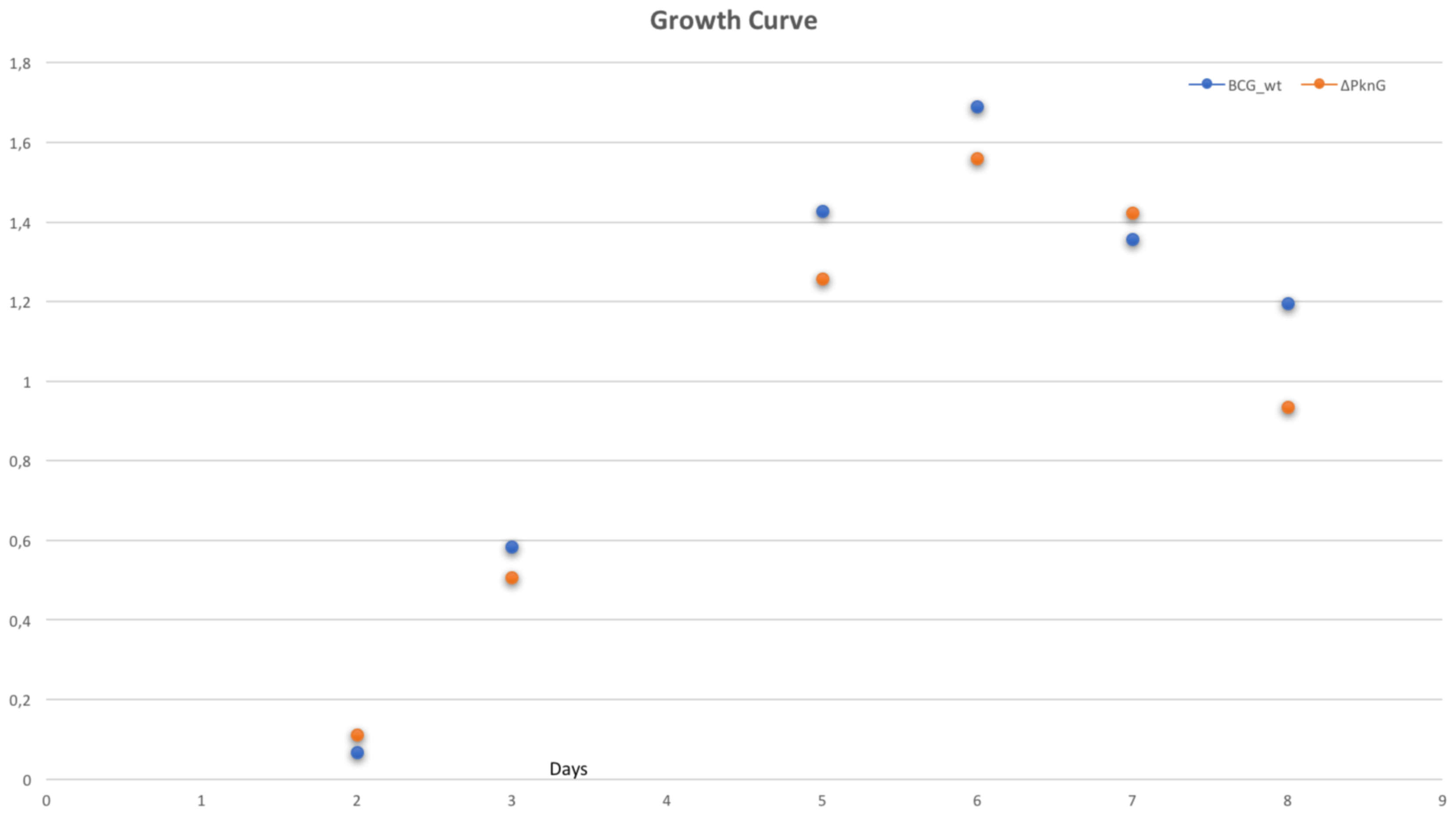

Supplementary Figure 1(a) Growth curves measured by OD_{600} of the *M. bovis* BCG strains used in this study.

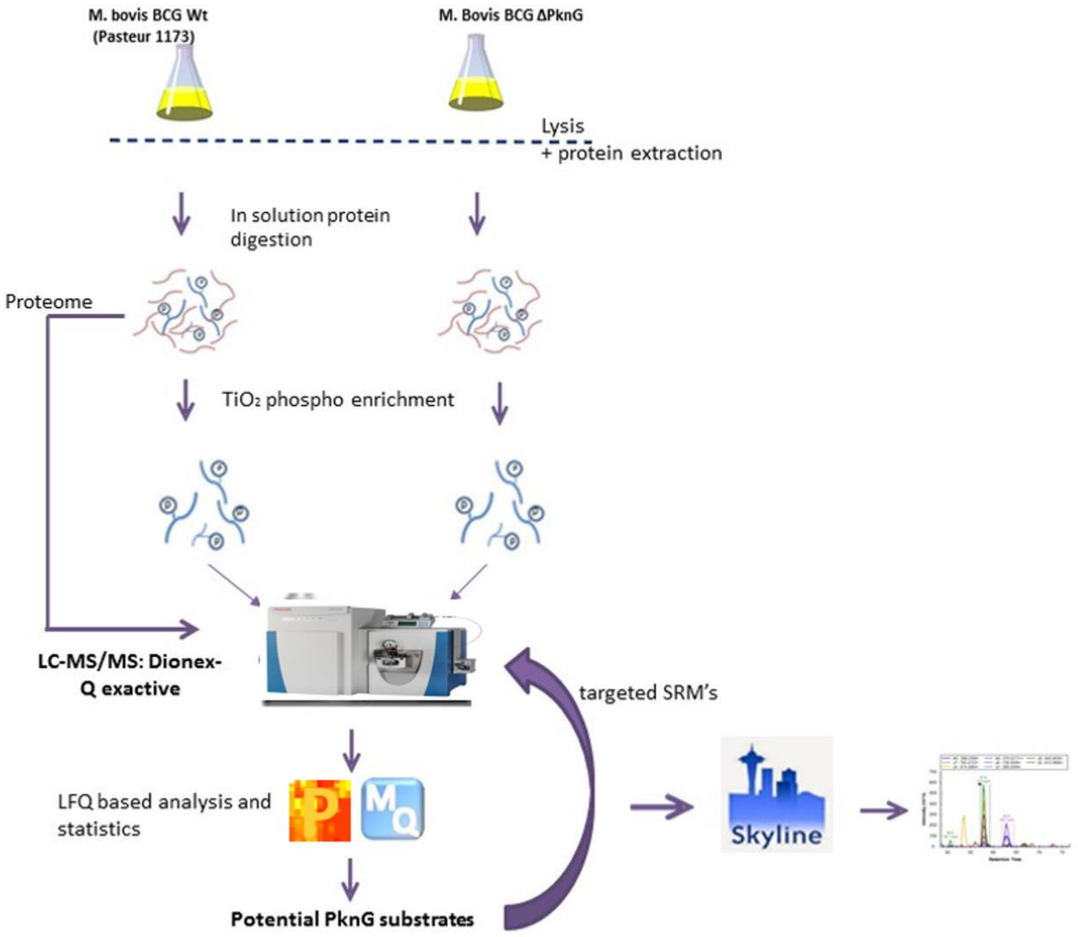

Supplementary Figure1: Experimental procedures in this study. Briefly, exponentially growing cells of *Mycobacterium bovis* BCG Wt and PknG knock-out mutants were harvested, lysed. ProtC. eins were digested in solution after precipitation with Methanol/chloroform. Three rounds of TiO2 enrichment of phosphopeptides was carried out and measured on the QE. Data processing and analysis was done on Maxquant and Perseus. Targeted MS on peptides of interest was analysed on skyline

Raw file	Scan	Method	Score	m/z
160318_BCG_Wt2_F2_4ul	11777	FTMS; HCD	49.43	1091.79

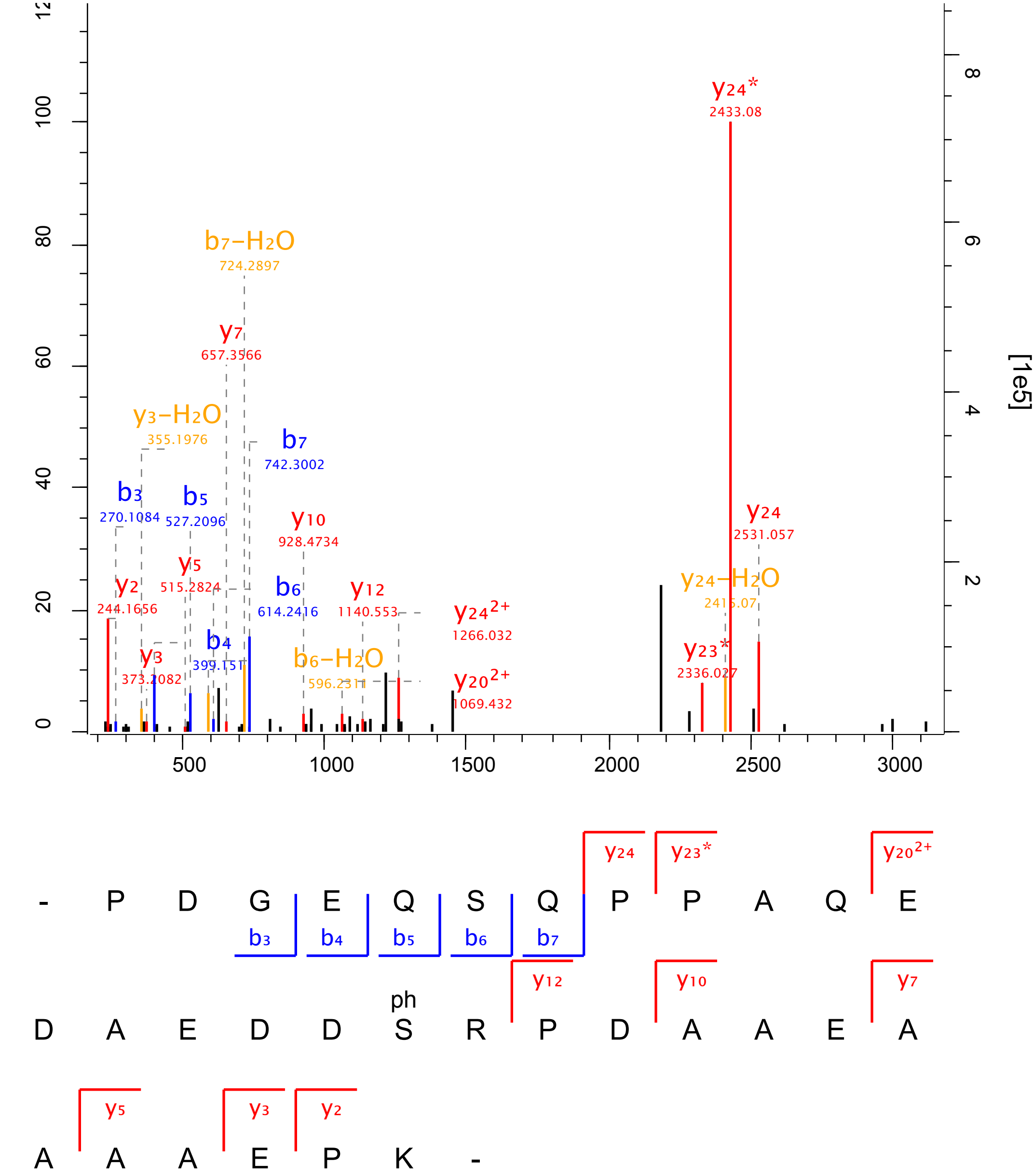

Raw file	Scan	Method	Score	m/z
160318_BCG_Wt3_F2_4ul	11017	FTMS; HCD	58.48	575.25

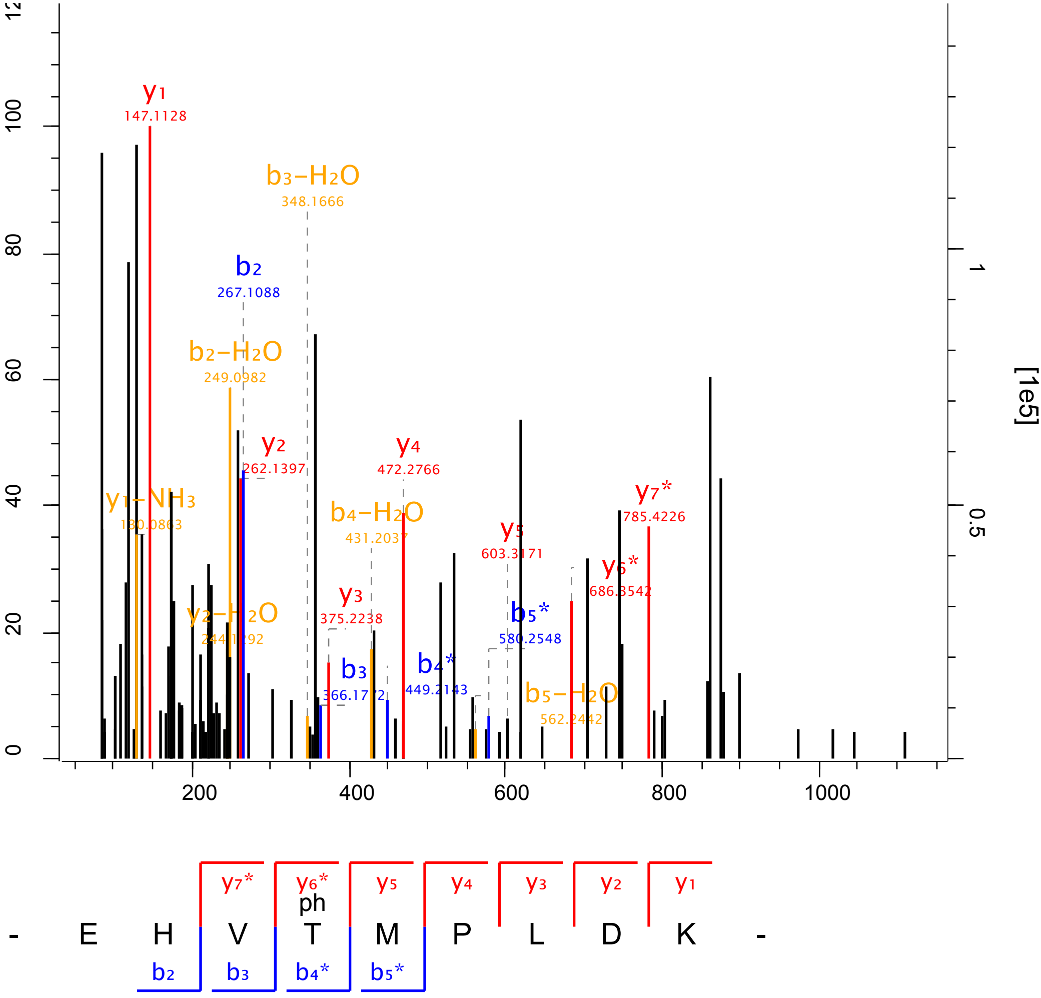

Raw file	Scan	Method	Score	m/z
160318_BCG_Wt3_F1_3ul	21629	FTMS; HCD	95.03	1257.93

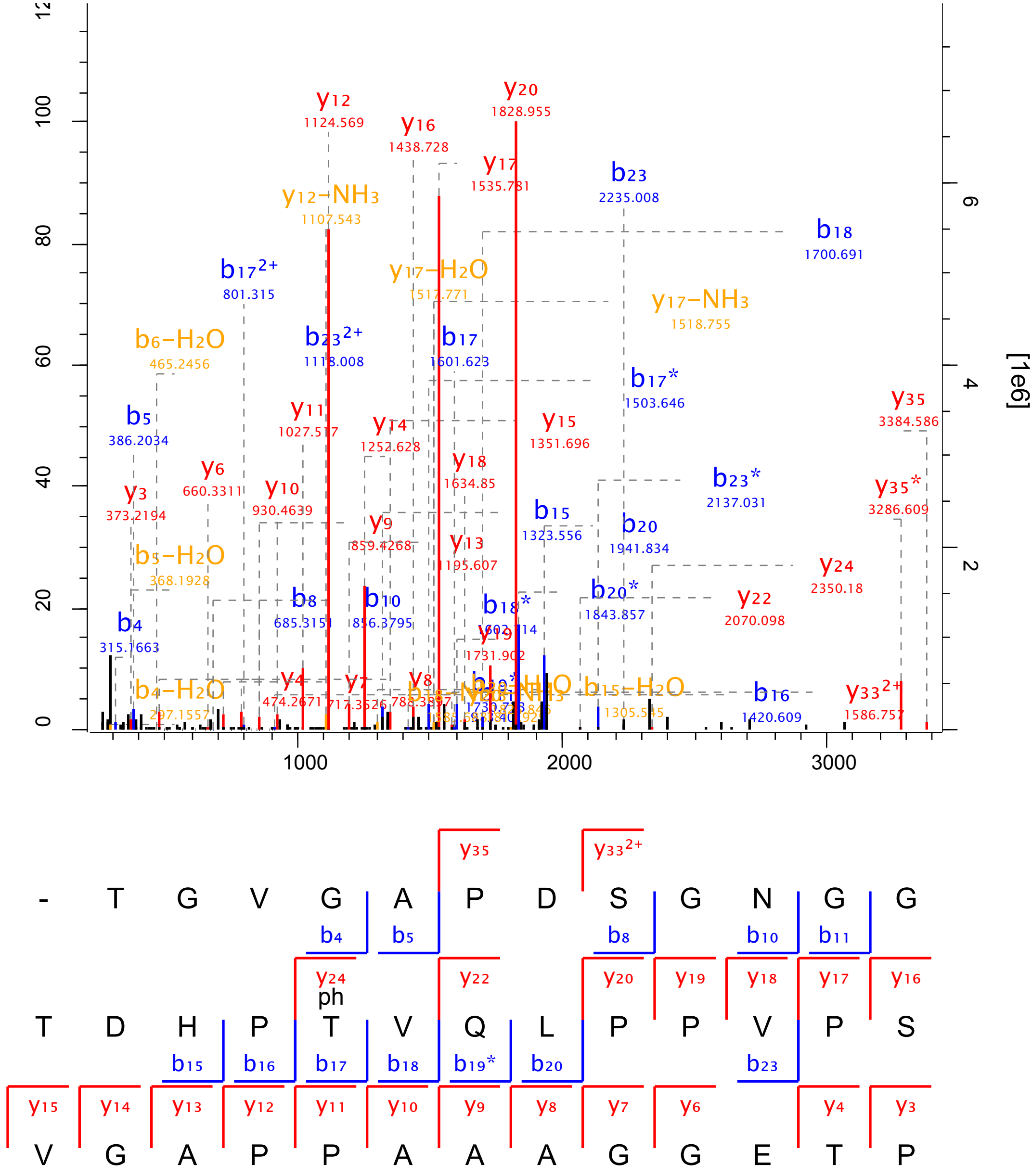

Raw file	Scan	Method	Score	m/z
160318_BCG_Wt2_F2_4ul	20114	FTMS; HCD	46.54	764.82

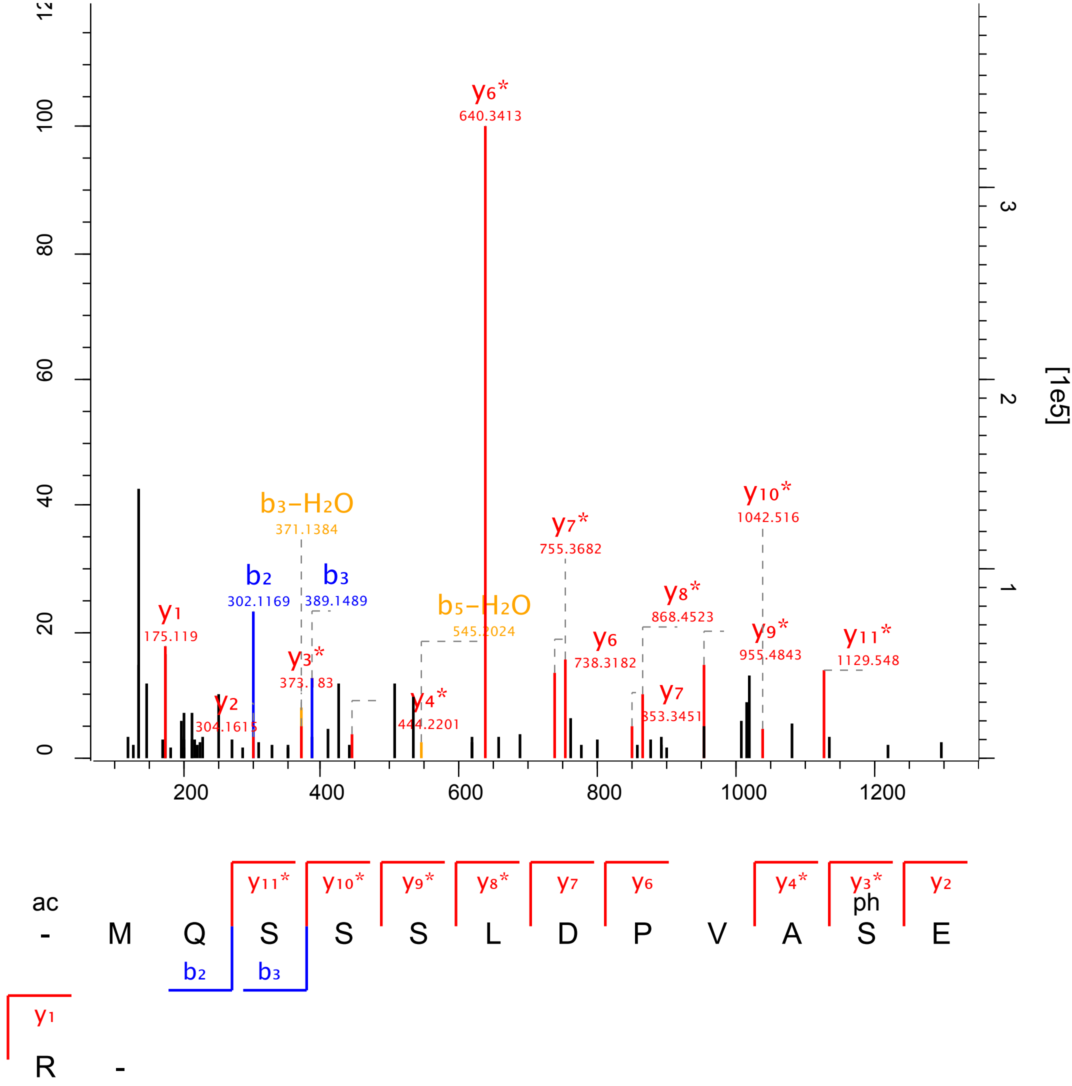

Raw file	Scan	Method	Score	m/z
160318_BCG_Wt3_F2_4ul	16235	FTMS; HCD	48.53	580.26

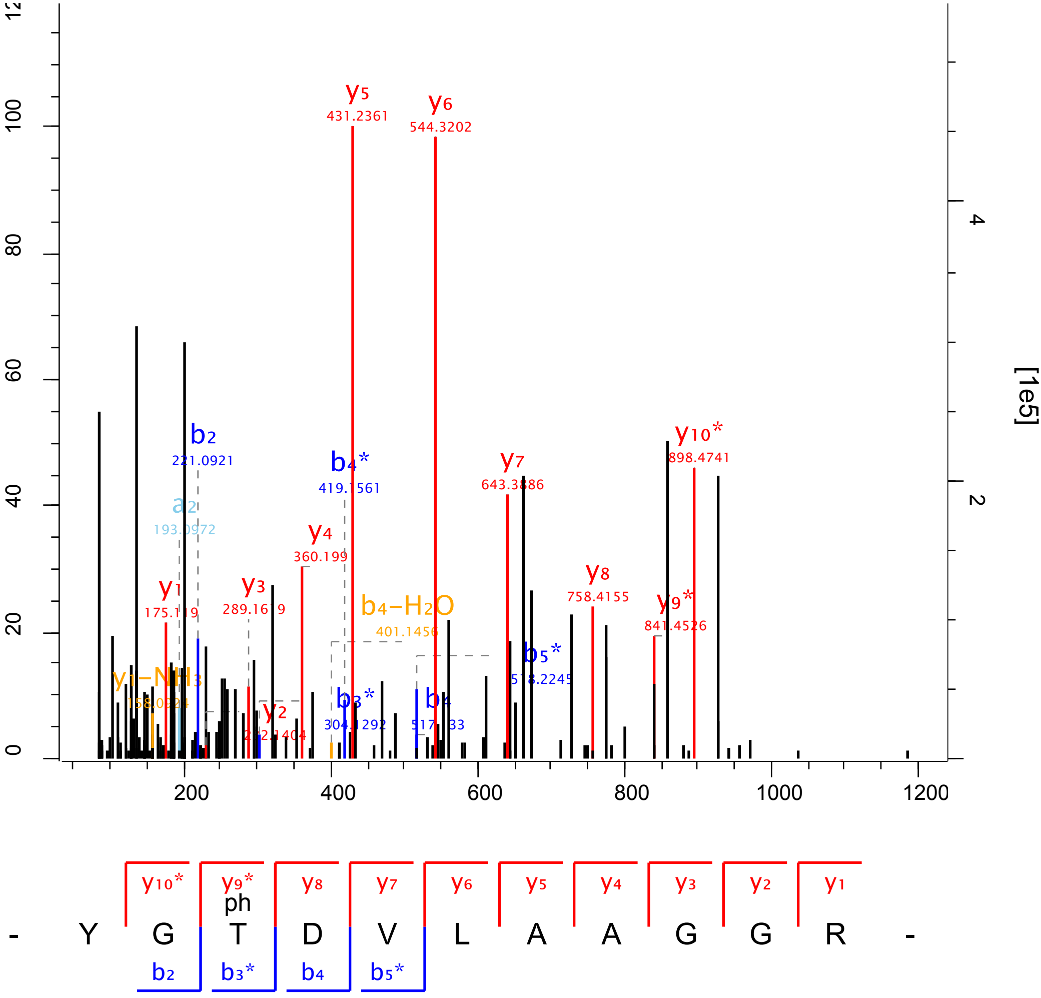

Raw file	Scan	Method	Score	m/z
160318_BCG_Wt4_F1_3ul	6174	FTMS; HCD	48.53	667.29

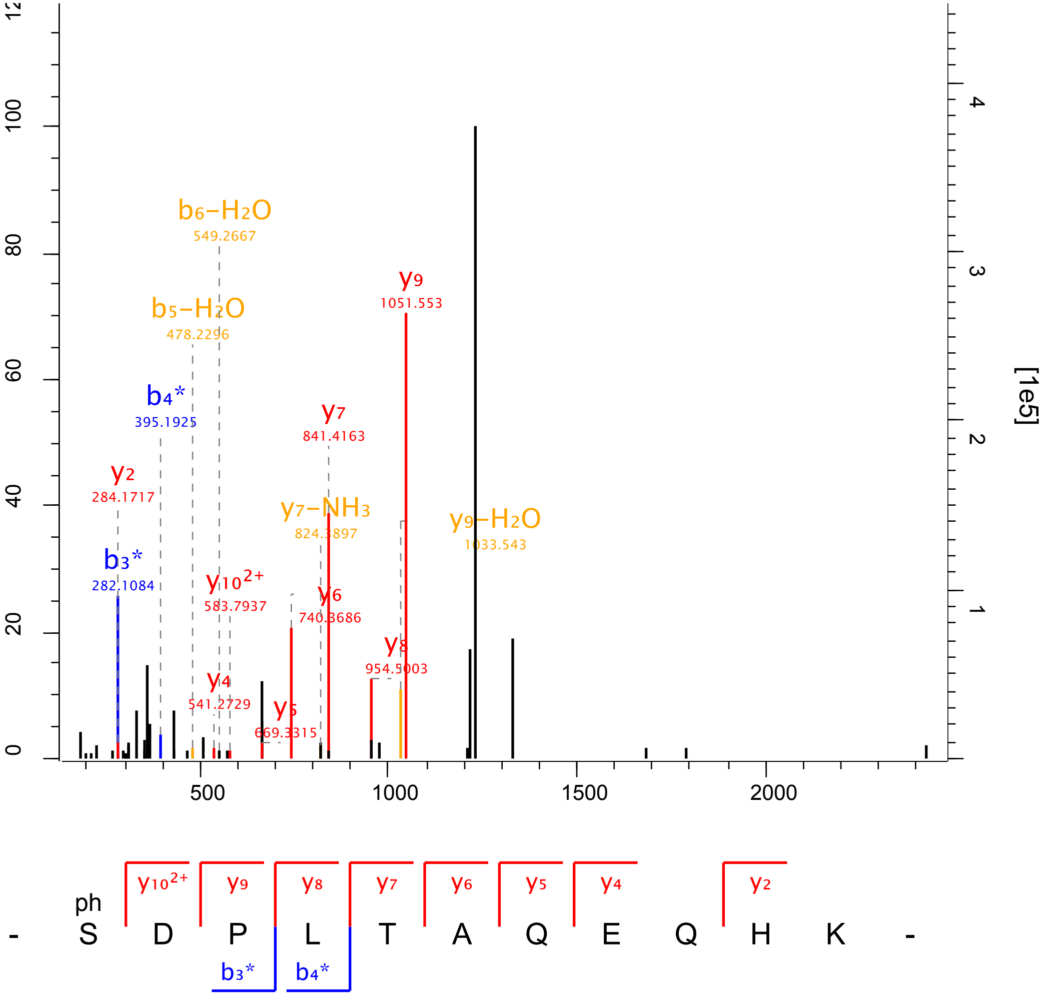

Raw file	Scan	Method	Score	m/z
160318_BCG_Wt3_F2_4ul	17704	FTMS; HCD	118.37	779.35

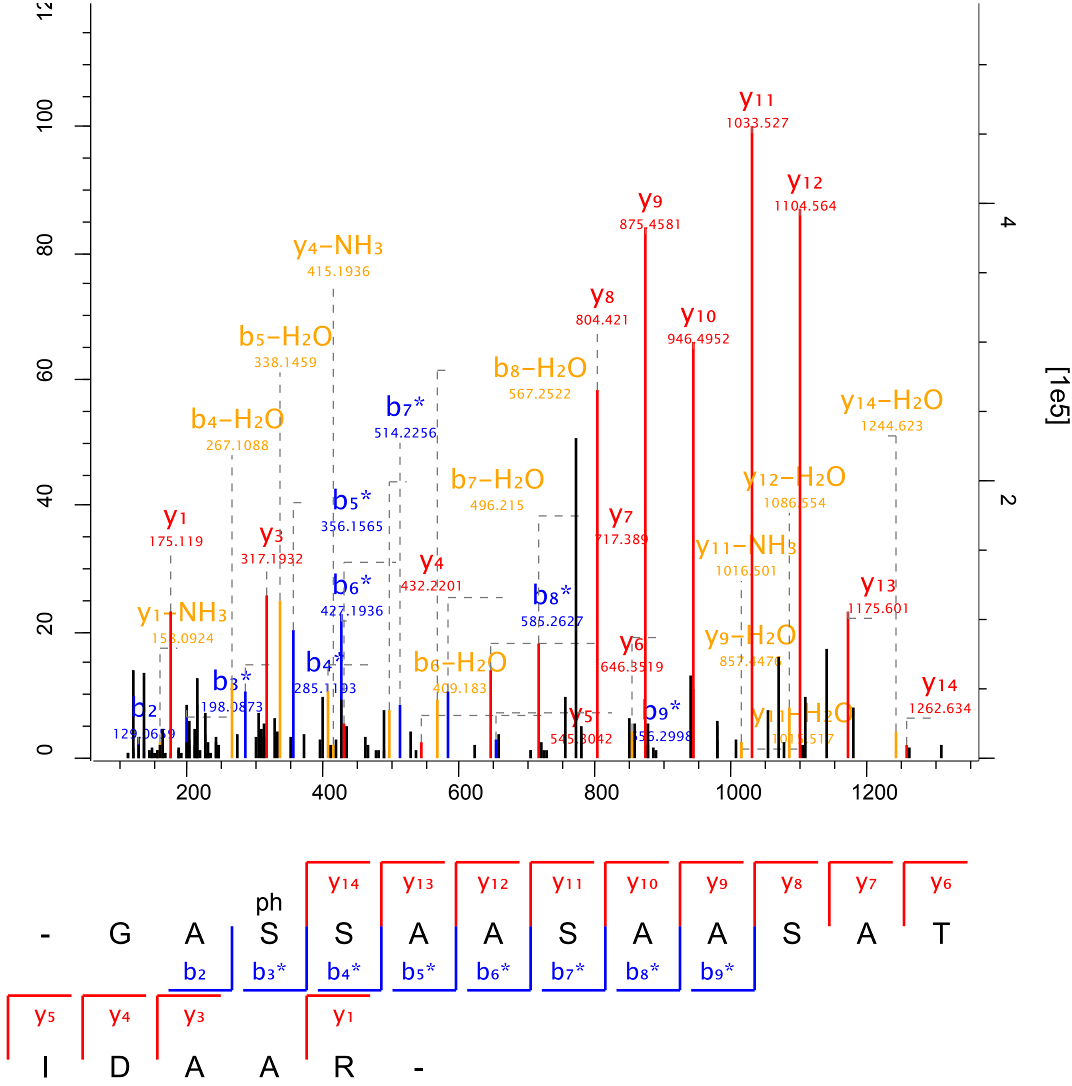

Raw file	Scan	Method	Score	m/z
160318_BCG_Wt2_F2_4ul	20618	FTMS; HCD	163.18	1082.95

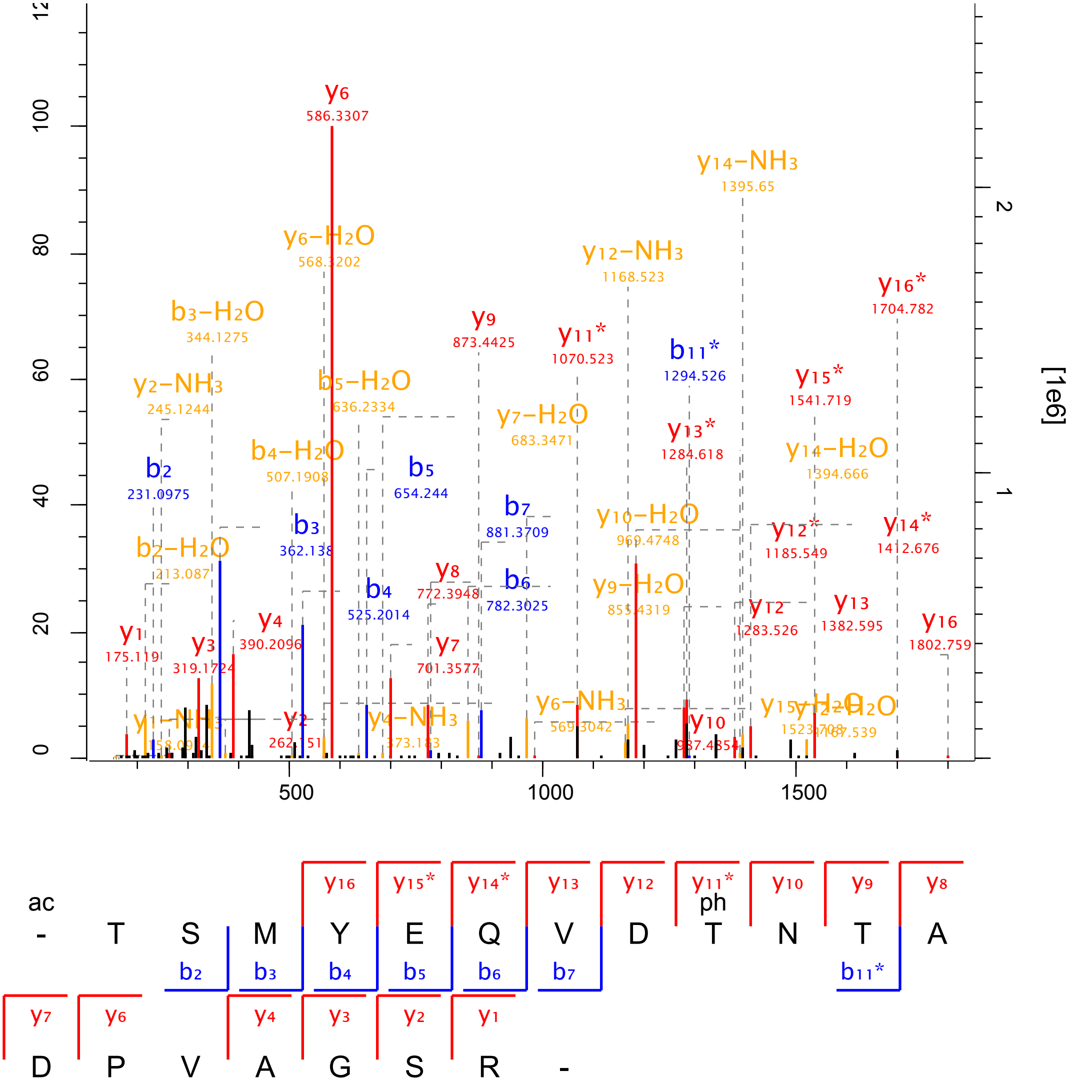

Raw file	Scan	Method	Score	m/z
160318_BCG_Wt2_F2_4ul	4832	FTMS; HCD	107.97	641.74

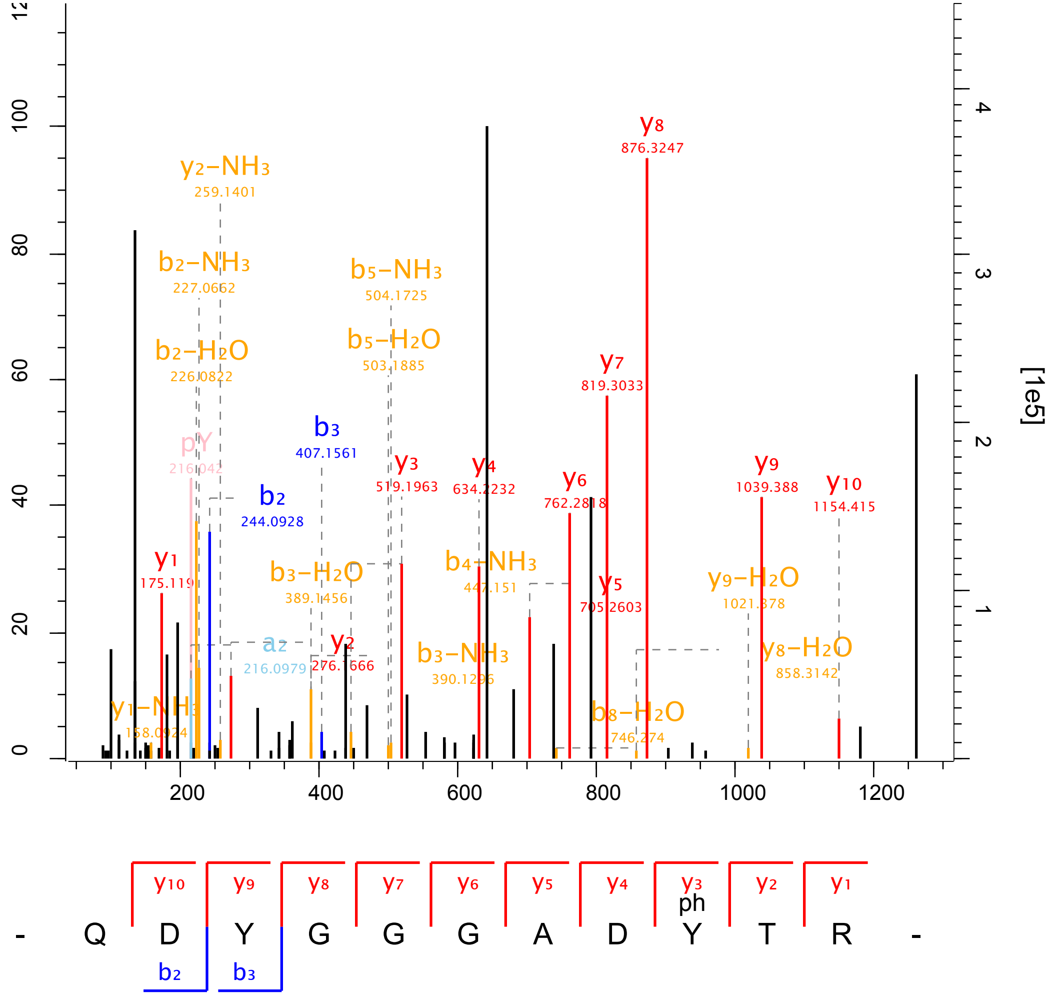

Raw file	Scan	Method	Score	m/z
160318_BCG_Wt2_F2_4ul	15494	FTMS; HCD	87.79	1217.03

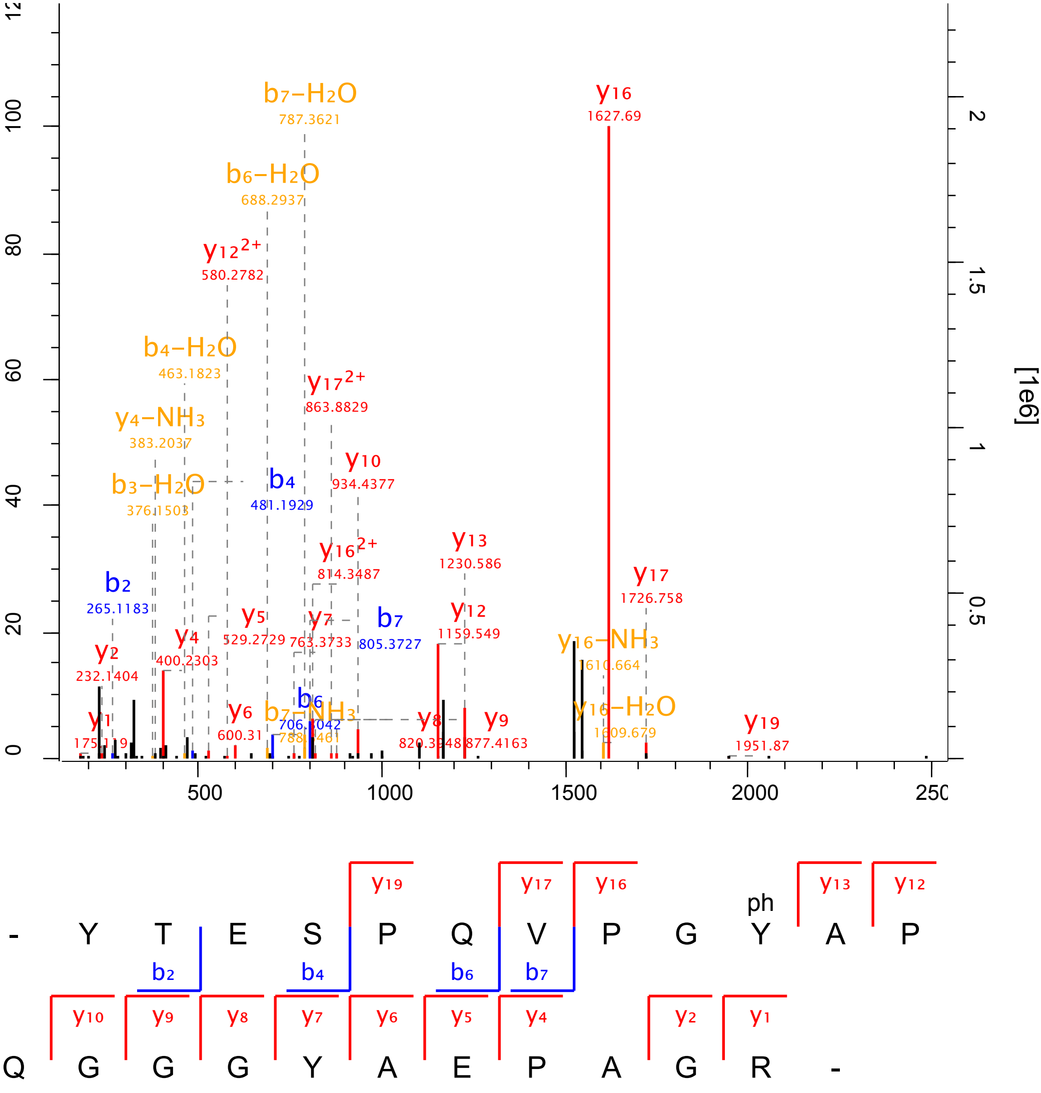

Raw file	Scan	Method	Score	m/z
160318_BCG_Wt3_F2_4ul	21957	FTMS; HCD	39.98	942.49

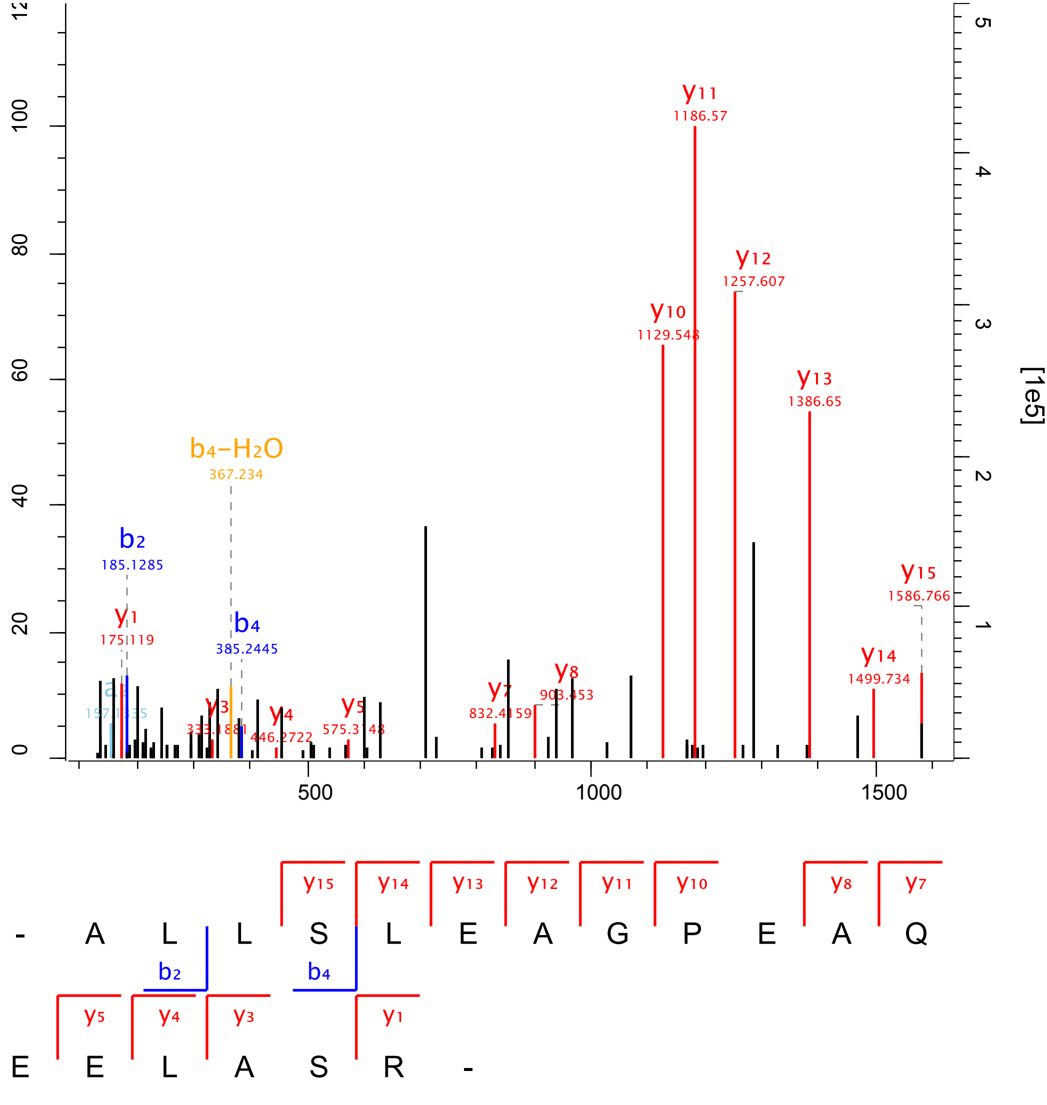

Raw file	Scan	Method	Score	m/z
160318_PknG2_F1_3ul	17497	FTMS; HCD	119.45	692.82

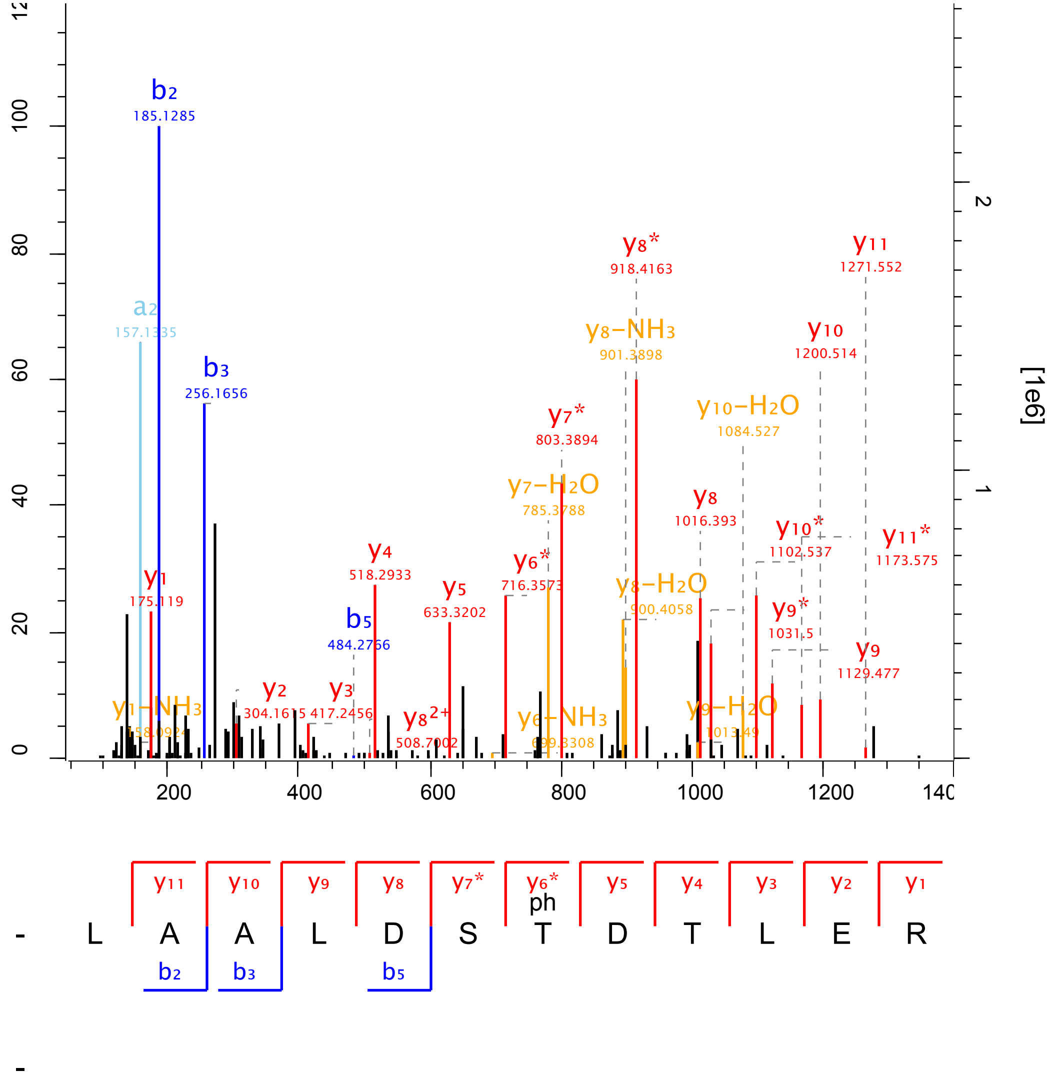

Raw file	Scan	Method	Score	m/z
160318_BCG_Wt3_F2_4ul	20845	FTMS; HCD	44.79	876.86

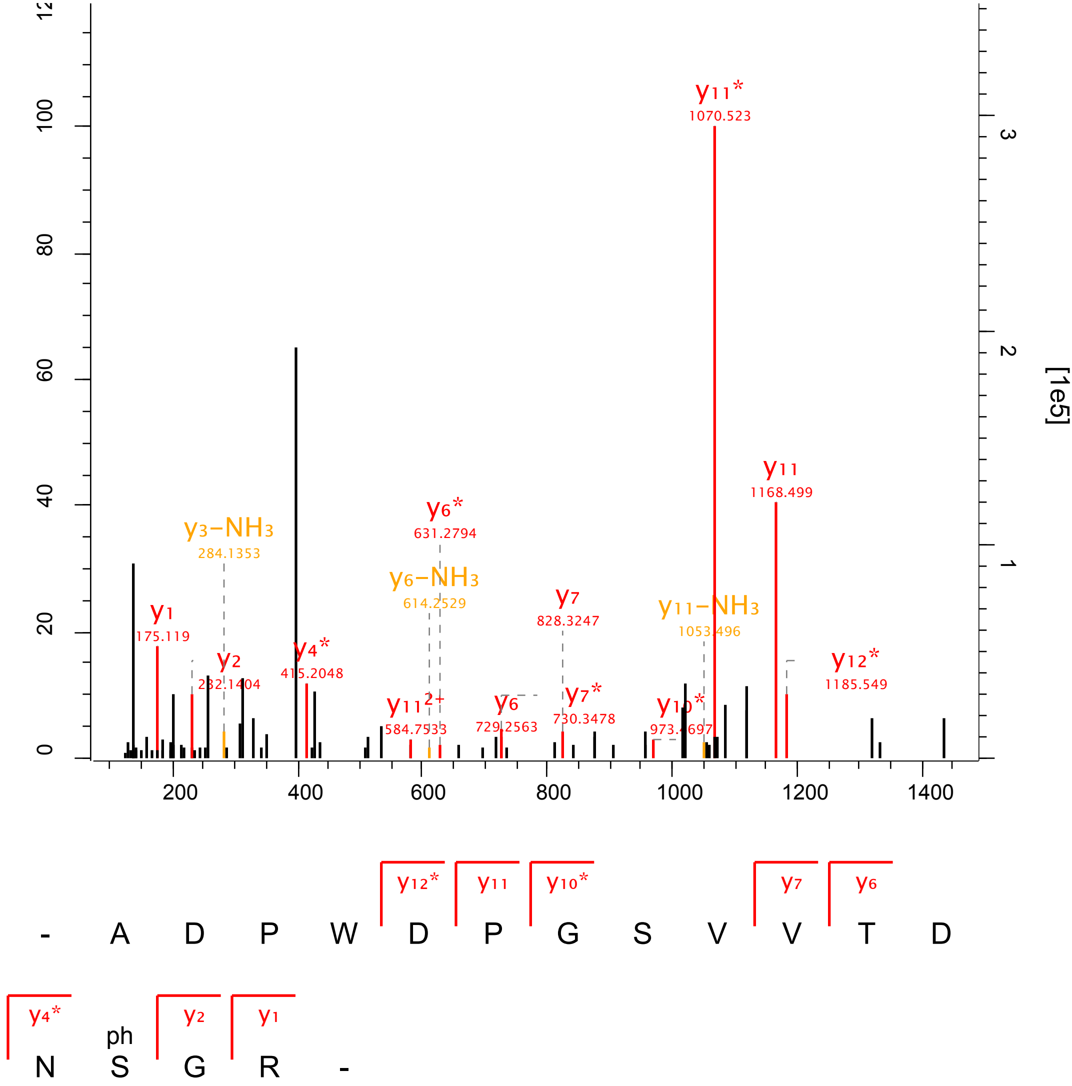

Raw file	Scan	Method	Score	m/z
160318_BCG_Wt4_F4_4ul	4657	FTMS; HCD	56.01	427.7

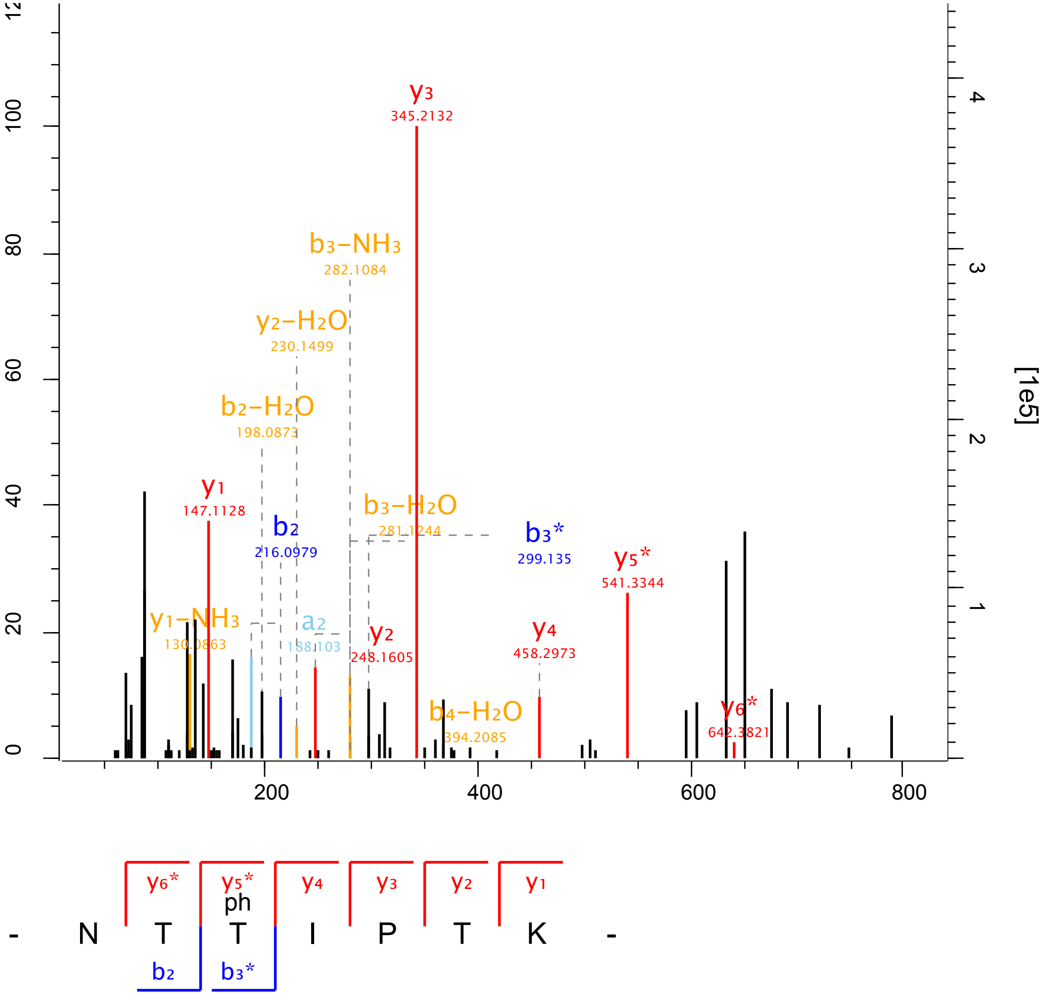

Raw file	Scan	Method	Score	m/z
160318_BCG_Wt4_F1_3ul	7139	FTMS; HCD	40.72	372.2

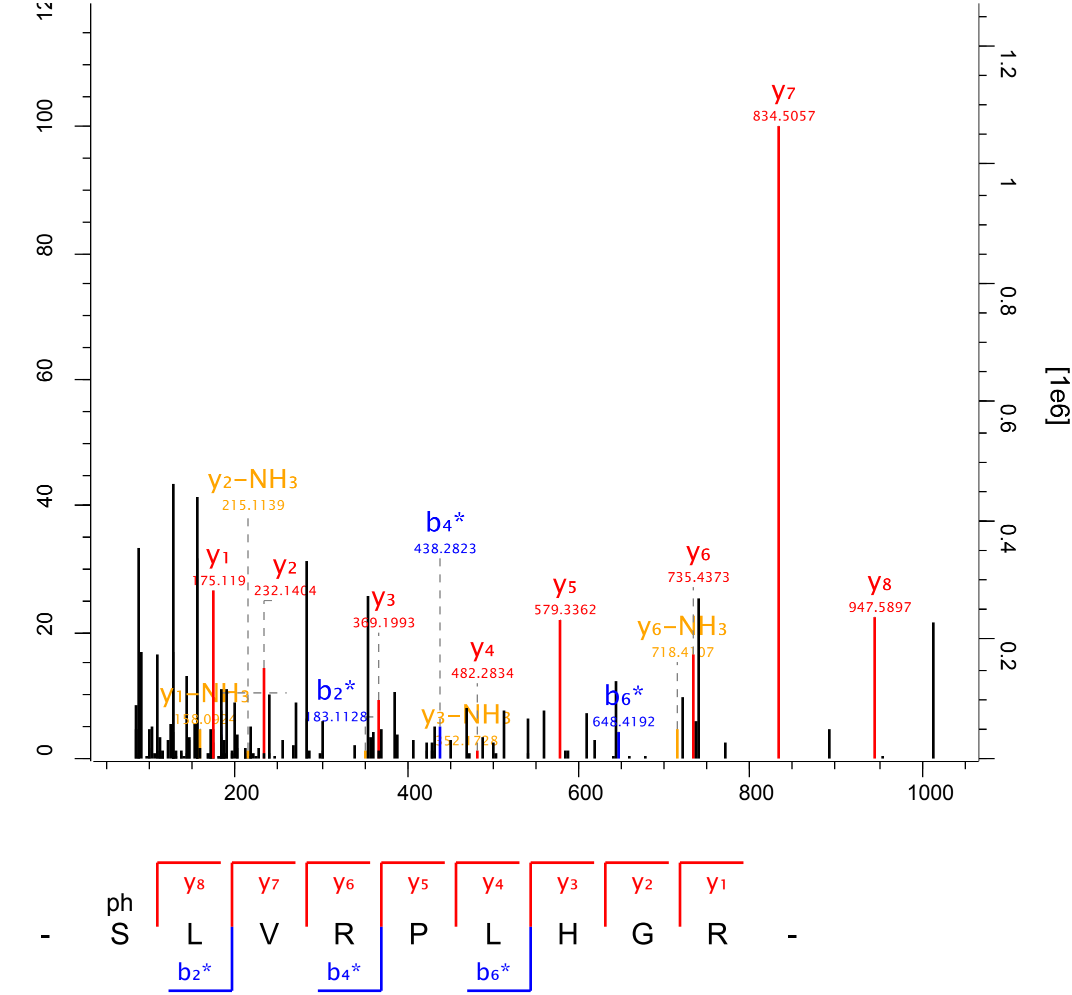

Raw file	Scan	Method	Score	m/z
160318_BCG_Wt2_F2_4ul	17481	FTMS; HCD	61.5	692.79

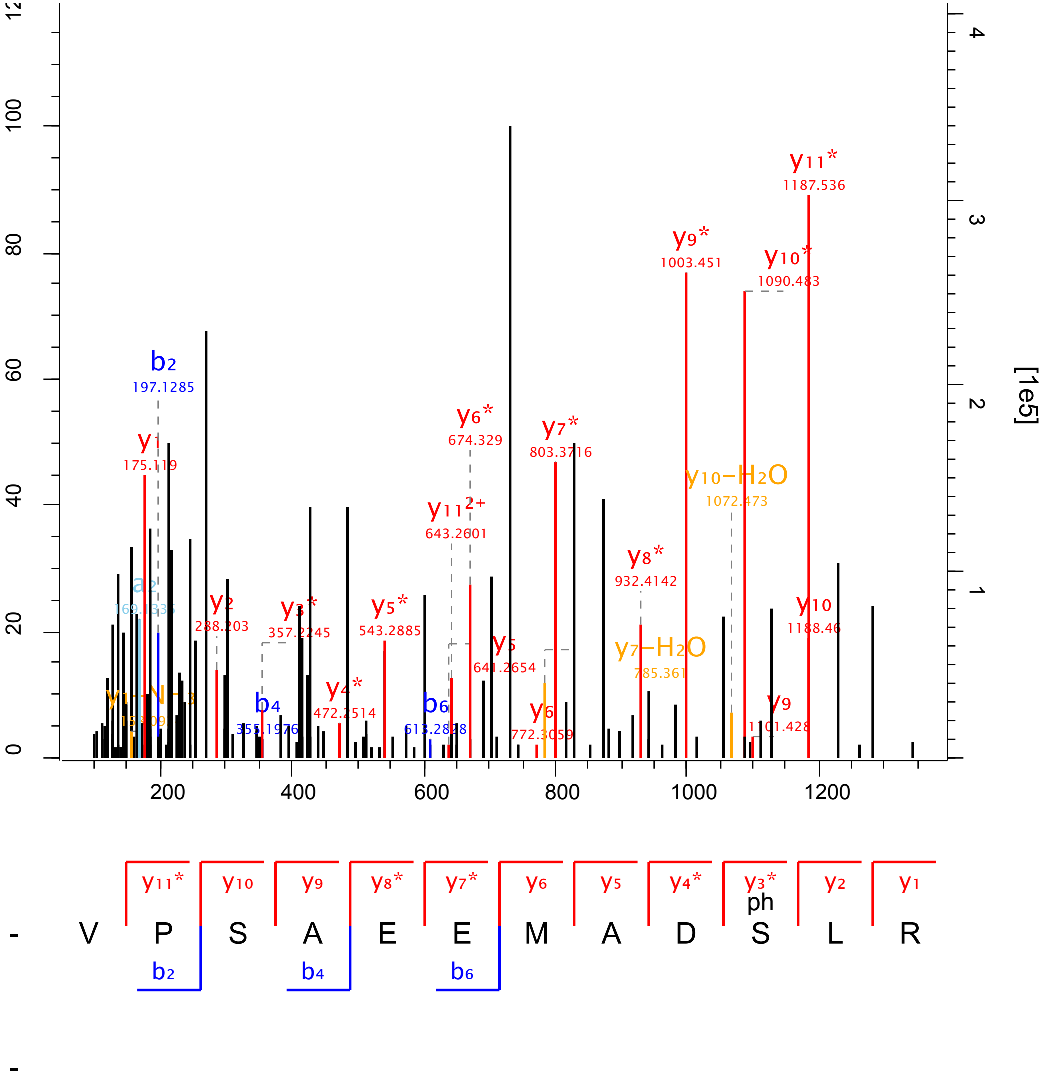

Raw file	Scan	Method	Score	m/z
160318_BCG_Wt3_F1_3ul	21629	FTMS; HCD	95.03	1257.93

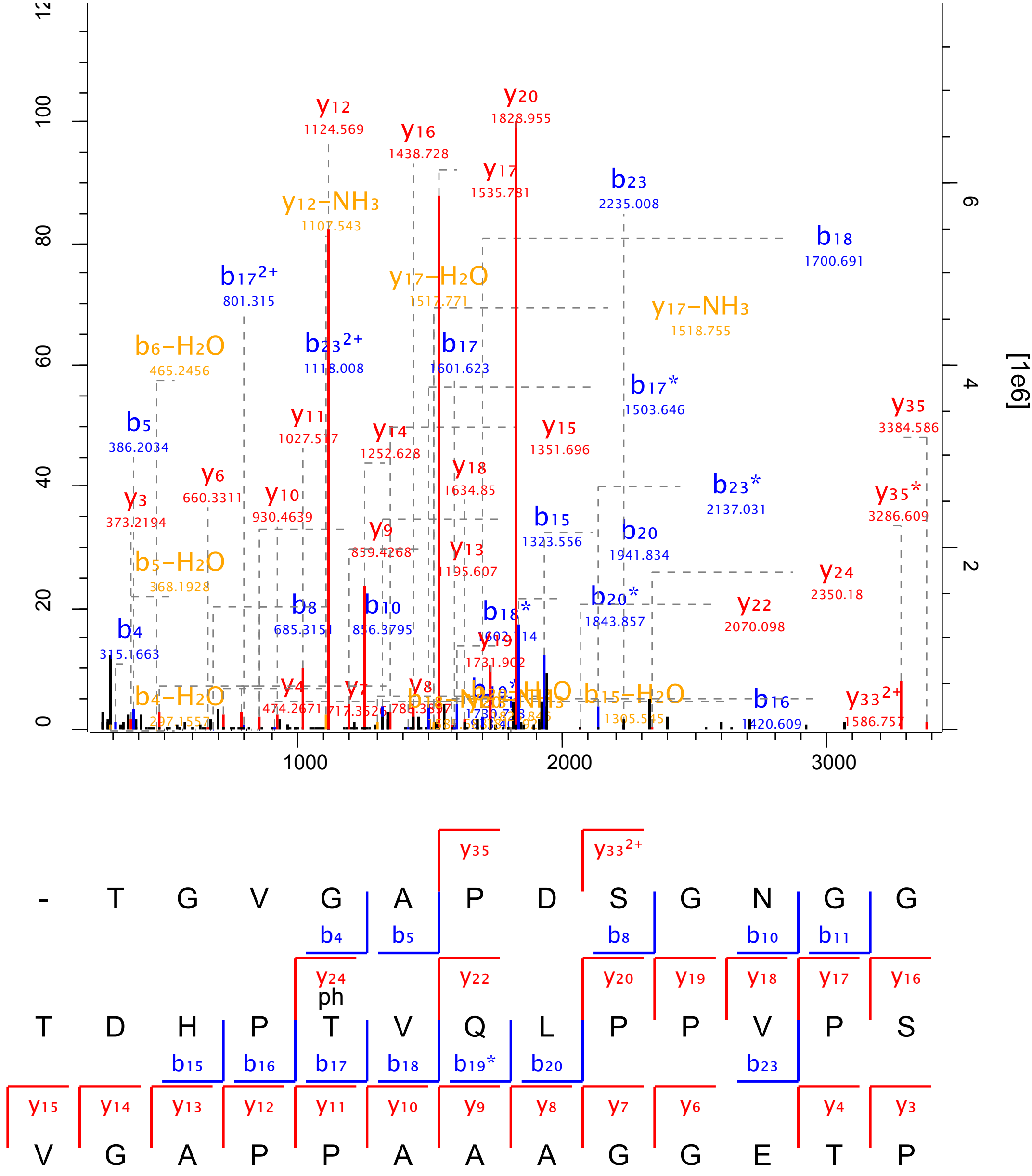

Raw file	Scan	Method	Score	m/z
160318_BCG_Wt3_F2_4ul	17704	FTMS; HCD	118.37	779.35

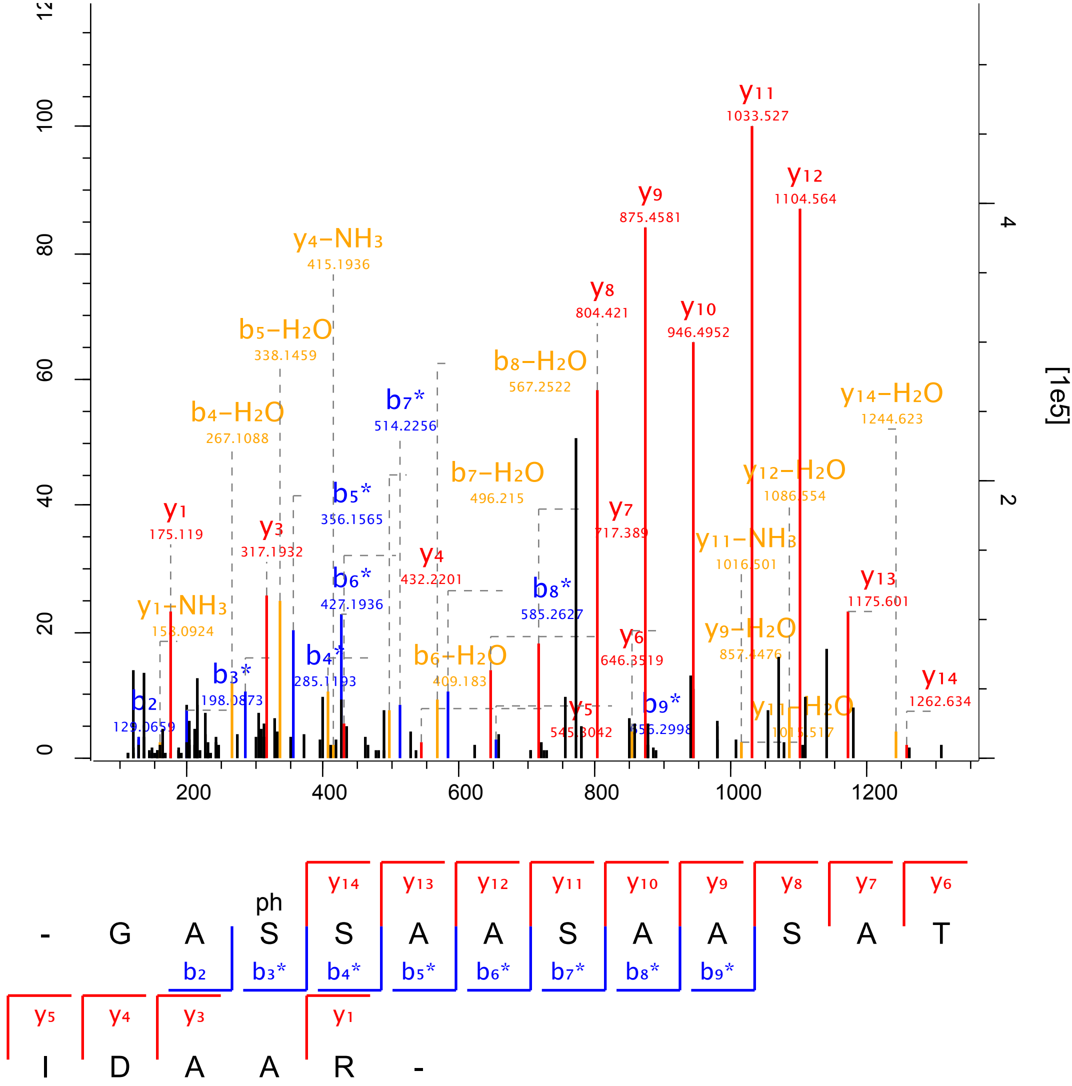

Raw file	Scan	Method	Score	m/z
160318_BCG_Wt3_F1_3ul	1600	FTMS; HCD	50.39	533.24

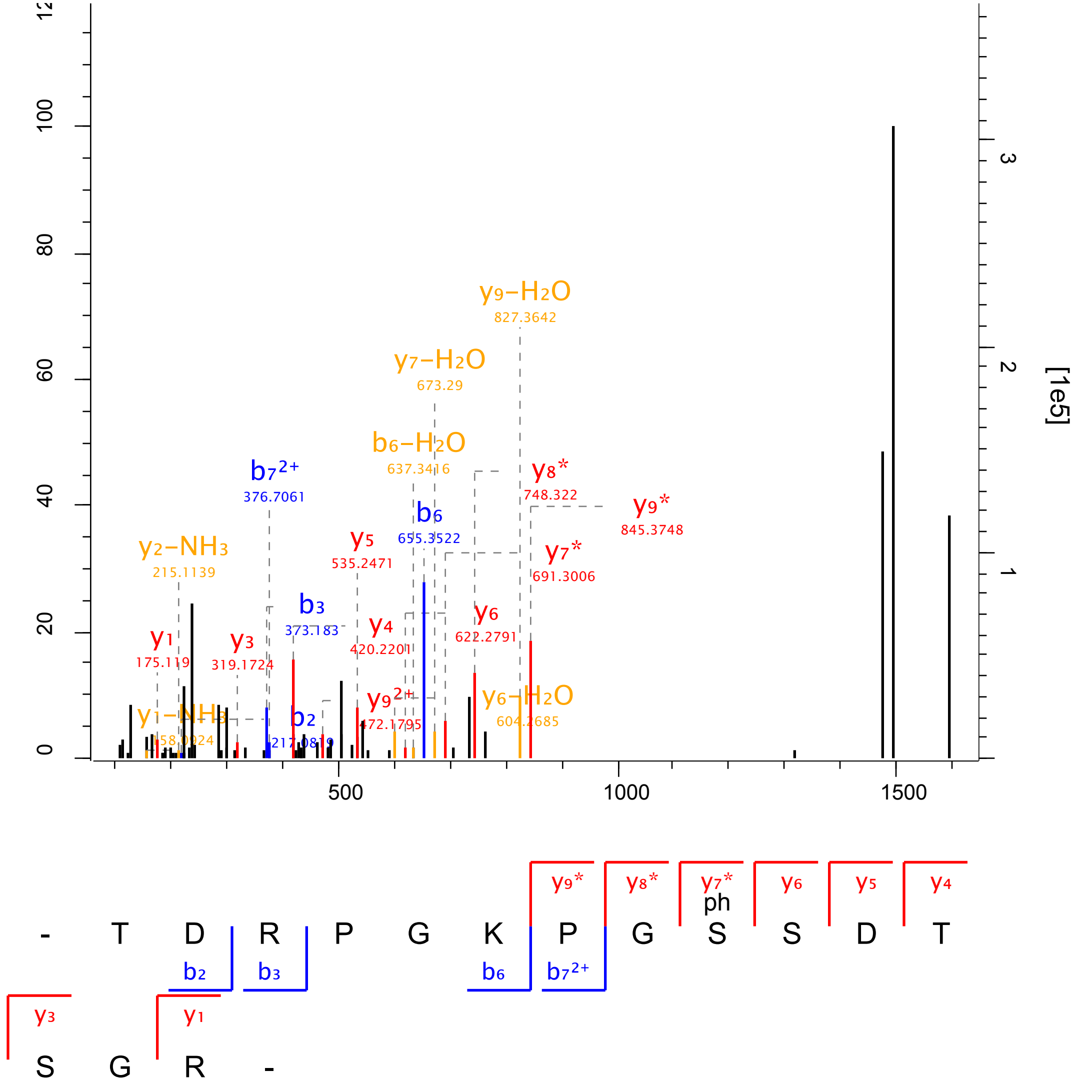

Raw file	Scan	Method	Score	m/z
160318_BCG_Wt2_F2_4ul	21650	FTMS; HCD	81.8	1148.02

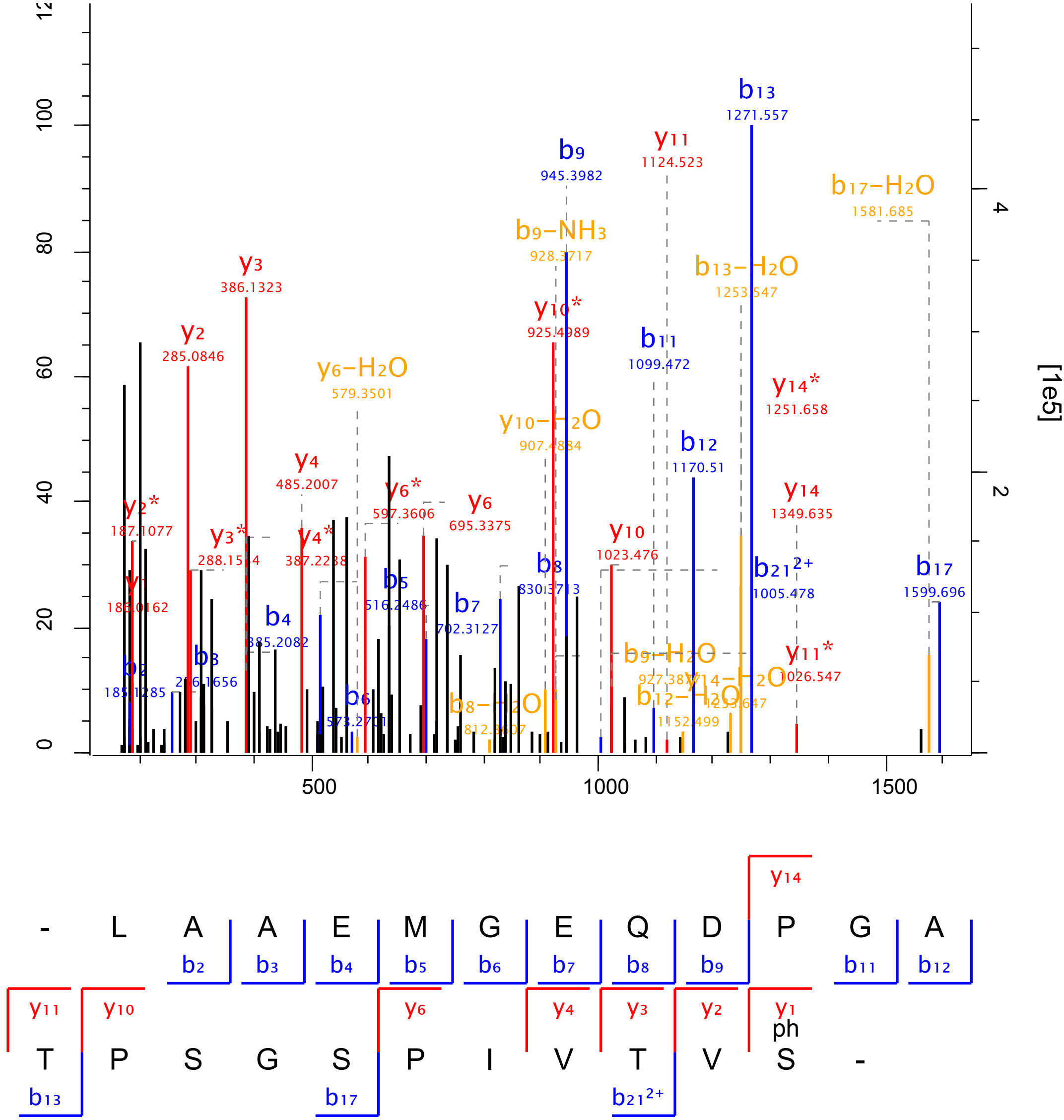

Raw file	Scan	Method	Score	m/z
160318_BCG_Wt2_F2_4ul	20346	FTMS; HCD	64.4	1393.12

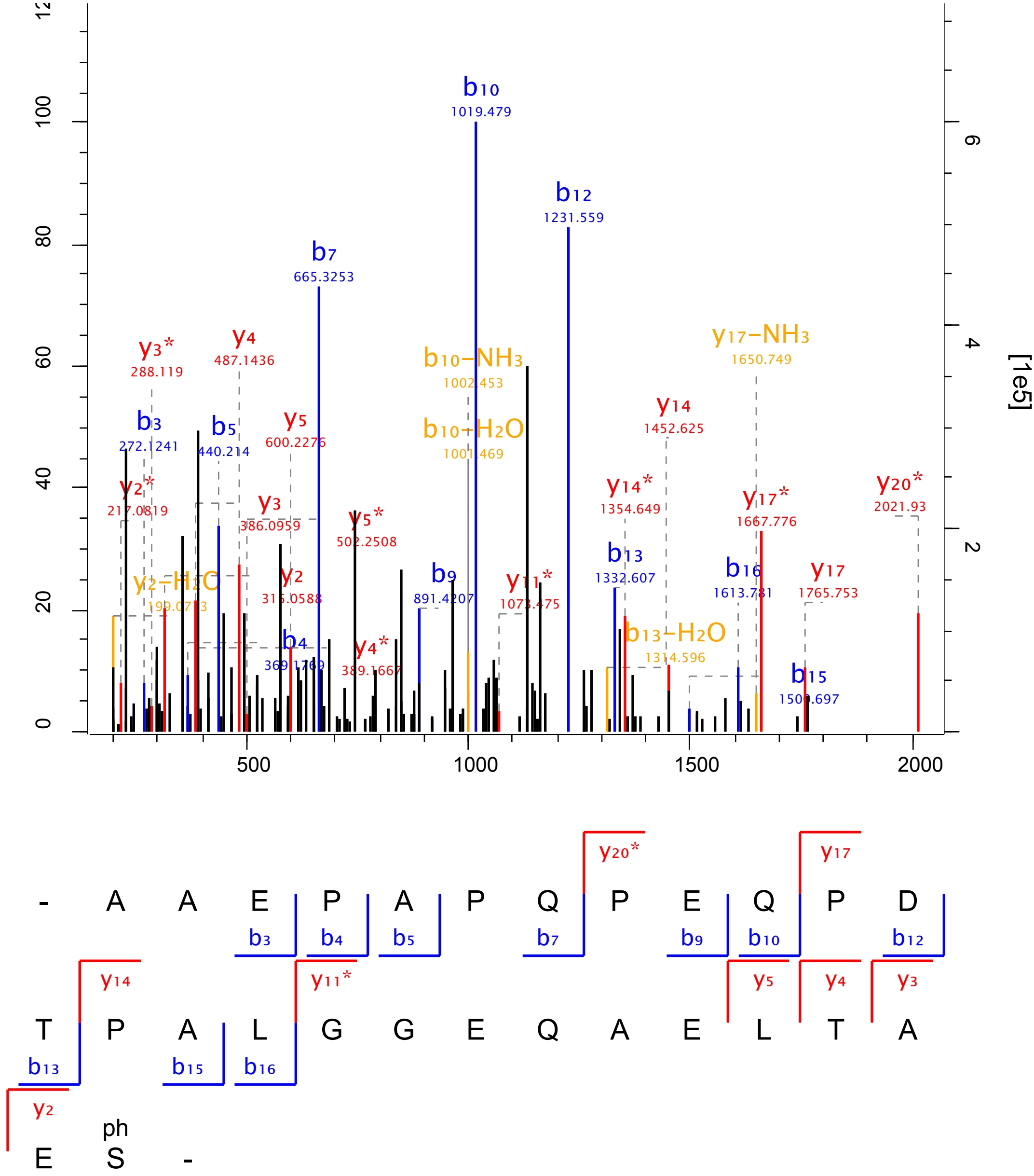

Supplement: Supplemental Data [file supp_RA118.000705_136253_0_supp_81858_p4lpdm.pdf]
